# AA15 lytic polysaccharide monooxygenase is required for efficient chitinous cuticle turnover during insect molting

**DOI:** 10.1038/s42003-022-03469-8

**Published:** 2022-05-31

**Authors:** Mingbo Qu, Xiaoxi Guo, Shuang Tian, Qing Yang, Myeongjin Kim, Seulgi Mun, Mi Young Noh, Karl J. Kramer, Subbaratnam Muthukrishnan, Yasuyuki Arakane

**Affiliations:** 1 School of Bioengineering, Dalian University of Technology, 116024 Dalian, China; 2 State Key Laboratory for Biology of Plant Diseases and Insect Pests, Institute of Plant Protection, Chinese Academy of Agricultural Sciences, 100193 Beijing, China; 3 Shenzhen Branch, Guangdong Laboratory of Lingnan Modern Agriculture, Genome Analysis Laboratory of the Ministry of Agriculture and Rural Affairs, Agricultural Genomics Institute at Shenzhen, Chinese Academy of Agricultural Sciences, 518120 Shenzhen, China; 4 Department of Applied Biology, Chonnam National University, Gwangju, 61186 South Korea; 5 Department of Forest Resources, AgriBio Institute of Climate Change Management, Chonnam National University, Gwangju, 61186 South Korea; 6 Department of Biochemistry and Molecular Biophysics, Kansas State University, Manhattan, KS 66506 USA

**Keywords:** Development, Developmental biology

## Abstract

Microbial lytic polysaccharide monooxygenases (LPMOs) catalyze the oxidative cleavage of crystalline polysaccharides including chitin and cellulose. The discovery of a large assortment of LPMO-like proteins widely distributed in insect genomes suggests that they could be involved in assisting chitin degradation in the exoskeleton, tracheae and peritrophic matrix during development. However, the physiological functions of insect LPMO-like proteins are still undetermined. To investigate the functions of insect LPMO15 subgroup I-like proteins (LPMO15-1s), two evolutionarily distant species, *Tribolium castaneum* and *Locusta migratoria*, were chosen. Depletion by RNAi of *T. castaneum TcLPMO15-1* caused molting arrest at all developmental stages, whereas depletion of the *L. migratoria LmLPMO15-1*, prevented only adult eclosion. In both species, LPMO15-1-deficient animals were unable to shed their exuviae and died. TEM analysis revealed failure of turnover of the chitinous cuticle, which is critical for completion of molting. Purified recombinant LPMO15-1-like protein from *Ostrinia furnacalis* (r*Of*LPMO15-1) exhibited oxidative cleavage activity and substrate preference for chitin. These results reveal the physiological importance of catalytically active LPMO15-1-like proteins from distant insect species and provide new insight into the enzymatic mechanism of cuticular chitin turnover during molting.

## Abstract (web summary)

Microbial lytic polysaccharide monooxygenases (LPMOs) catalyze the cleavage of chitin and cellulose. Here, characterisation of LPMO15-1-like proteins from insect reveal their catalytic activity and physiological importance for insect development.

## Introduction

Insect cuticle consists of morphologically distinct tissue layers including the outermost envelope, epicuticle, and innermost procuticle (exocuticle and endocuticle), the latter consisting of a large number of horizontally oriented chitin-protein rich laminae^1–3^. To accommodate growth, insects periodically replace their cuticle in a process involving ecdysis and molting. The turnover of chitin in the cuticle by enzymes presents several challenges because of the crystallinity of the anti-parallel chitin chains and potential covalent cross-linking to cuticular proteins. The roles of chitinolytic enzymes in molting are relatively well understood^4–6^. However, the functions of insect lytic polysaccharide monooxygenases (LPMOs) that have the potential to digest chitin are less well studied and thus, are the focus of this research.

LPMOs belong to a class of proteins that assist in the degradation of crystalline polymeric carbohydrate substrates (EC 1.14.99.53/54/55/56; https://www.qmul.ac.uk/sbcs/iubmb/enzyme/EC1/1499.html). They are copper-dependent enzymes that catalyze oxidative cleavage of glycosidic bonds in polysaccharides such as chitin, cellulose, and starch in the presence of a reductant and oxygen^7^. LPMOs have been identified in a wide range of organisms including bacteria, fungi, viruses, archaea and algae, as well as in higher animals such as cnidaria, mollusks, and arthropods^7–11^. They are classified in the Carbohydrate-Active EnZymes database (CAZy) into eight auxiliary activity (AA) families including AA9 (formerly glycosyl hydrolase family 61, GH61), AA10 (formerly carbohydrate-binding module family 33, CBM33), AA11 and AA13-AA17 enzymes^8,12–19^. In insects the gene family encoding LPMO-like proteins has been identified recently and determined to belong to the AA15 family (LPMO15)^8^. In addition, insect LPMO15s of this family could be further classified into several subgroups based on phylogenetic analysis and the presence of additional motifs^8,20^.

Bacterial LPMOs initially were identified as non-catalytic carbohydrate-binding proteins (CBP21 and CBM33), which enhanced polysaccharide degradation catalyzed by glycoside hydrolases^21^. However, Vaaje-Kolstad et al.^22^ demonstrated subsequently that the proteins CBP21 and CBM33A from *Serratia marcescens* and *Enterococcus faecalis*, respectively, are able to cleave glycosidic bonds of crystalline chitin in an oxidative fashion, creating new access points to the substrate for other effectors, such as chitinolytic enzymes. After these initial findings, other enzymatic properties such as substrate specificity and modes of action of LPMOs from microorganisms and viruses were examined extensively. Those studies demonstrated that LPMOs could cleave recalcitrant polysaccharides including chitin, cellulose, starch, xylan, pectin, and various other hemicelluloses, as well as soluble cello-oligosaccharides^14–16,23–29^. Only a few studies on the enzymatic properties of LPMO15-like proteins from insects have been reported so far. For example, Sabbadin et al.^8^ identified 23 genes encoding LPMO15-like proteins (*Td*LPMOs) in the transcriptome of the firebrat, *Thermobia domestica* (*Td*), which is an ancient insect species capable of digesting crystalline cellulose by using its own enzymes without the assistance of enzymes from microbial symbionts, with 21 of them (including *Td*AA15A and *Td*AA15B) present in the gut proteome. The recombinantly expressed *Td*AA15A protein, which is one of the most abundant LPMO15-like proteins from *T. domestica*, exhibited synergistic activity with enzymes of glycosyl hydrolase families GH6 (cellobiohydrolase) and GH18 (endochitinase) on the breakdown of cellulose and chitin, respectively. In contrast, another recombinant enzyme, *Td*AA15B, showed activity on crystalline chitin but not on cellulose. Similarly, two recombinant LPMO15s (*Cg*AA15a and *Cg*AA15b) from the lower termite, *Coptotermes gestroi*, catalyzed the oxidative cleavage of chitin, but not cellulose, xylan, xyloglucan, or starch^20^.

The primary role of bacterial LPMOs appears to be the degradation of polysaccharides as a nutrient carbon source. In addition, some studies of entomopathogenic and human pathogenic bacteria, oomycetous fungi and viruses have demonstrated their functional importance in pathogenicity. For instance, *Paneibacillus larvae* is a bacterial pathogen that causes a serious disease of honeybees, American Foulbrood (AFB). *Pl*CBP49 protein containing an LPMO domain (AA10 family) of *P. larvae* is critical for the degradation of the chitin-rich peritrophic matrix (PM) in the lining of the midgut of bee larvae, which is a vital step in the invasion of *P. larvae* during infection^30^. *Pseudomonas aeruginosa*, which exhibits multidrug-resistance, is an important opportunistic bacterial pathogen associated with nosocomial infections. CbpD, the tri-modular LPMO (AA10 family) of *P. aeruginosa*, is a chitin-oxidizing virulence factor that attenuates the terminal complement cascade in human serum and enhances survival of the bacterium in human blood^31^. *Phytophthora infestans* is a damaging crop pathogenic oomycete that infects both potato and tomato crops. A new family of AA17 LPMO from *P. infestans* has been reported to oxidatively cleave the backbone of pectin, playing an important role as virulence factors^19^. Similarly, spindles produced by entomopoxviruses (EVs) are cellular crystals of the LPMO-domain-containing protein, fusolin (AA10 family). This protein disrupts the host’s PM, leading to the greatly enhanced infectivity of EVs^32^.

As chitin is a major structural component of insect cuticle, tracheae and the PM^6^, LPMO-like proteins could be involved in the degradation of chitin in those tissues during development. However, the exact physiological functions of insect *LPMO15* genes are still not determined. In this study, we report on the physiological function of LPMO15-like proteins comprising one of the subgroups of insect LPMO15 (denoted as LPMO15-1) whose members have an AA15 LPMO catalytic domain and a conserved long C-terminal stretch of ~120 amino acids containing a cysteine-rich motif. Three economically important agricultural pests from three orders of Insecta were used as model species to investigate the function of insect LPMO15-1s. The holometabolous red flour beetle, *Tribolium castaneum* (Coleoptera), and hemimetabolous migratory locust, *Locusta migratoria* (Orthoptera), were utilized to study the biological function(s) of LPMO15-1s by RNA interference (RNAi), while an LPMO15-1 from the holometabolous Asian corn borer, *Ostrinia furnacalis* (Lepidoptera), was used to study the enzymatic properties of this class of enzymes. We provide experimental evidence for a functional importance of insect LPMO15-1s in the catabolic oxidative breakdown of cuticular chitin, which is also degraded by a mixture of molting fluid chitinases and β-*N*-acetylhexosaminidases^4–6^. This work provides new insight into the molecular mechanism underlying the vital process of cuticular chitin degradation in insect molting.

## Results

### Phylogenetic analysis of insect LPMOs

**Fig. 1:**
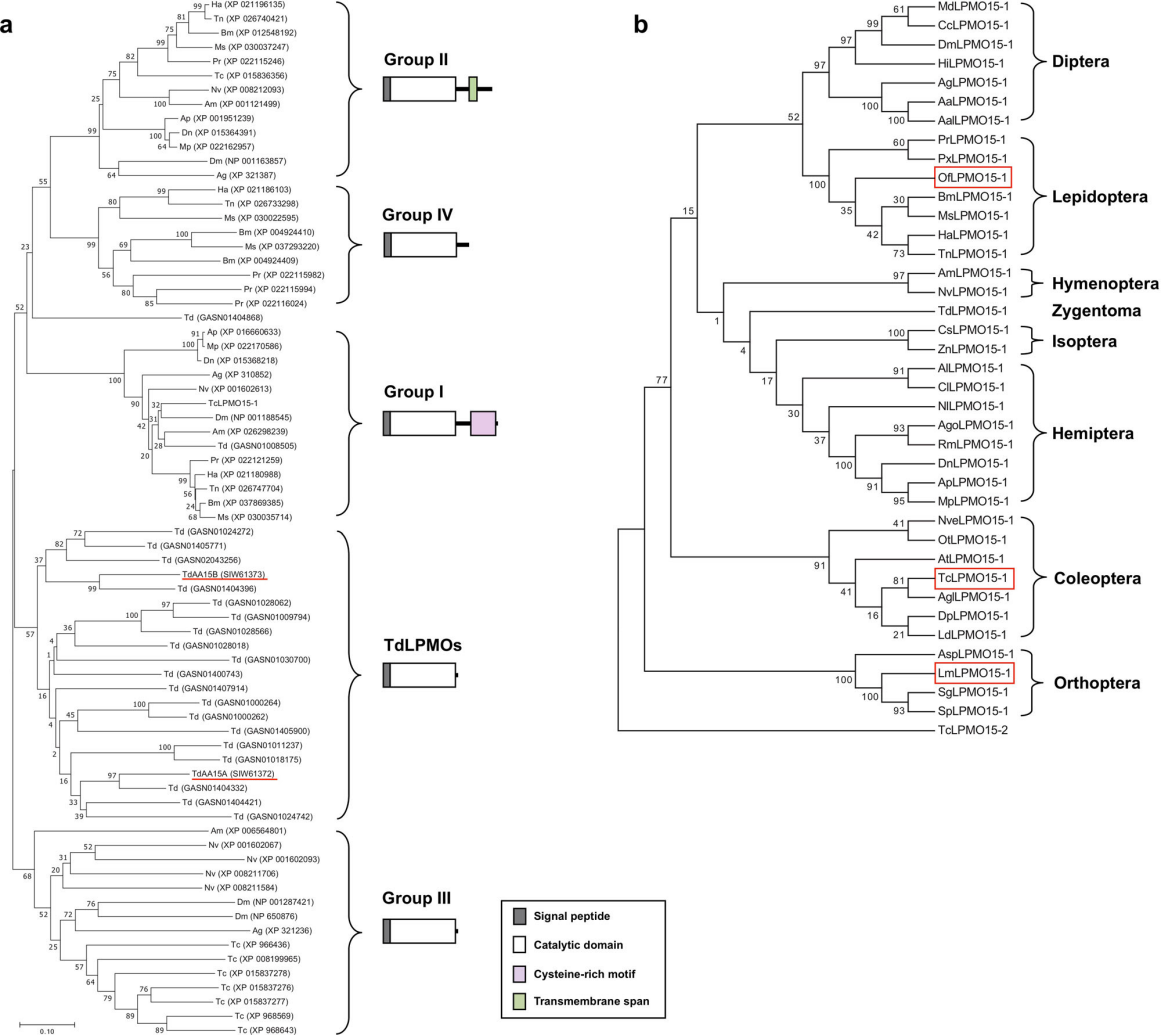
Phylogenetic analysis of insect LPMO15s. **a** Phylogenetic tree of putative LPMO15s from *T. castaneum* and other insect species whose genomes have been sequenced and annotated. Tc, *Tribolium castaneum*; Dm, *Drosophila melanogaster*; Ag, *Anopheles gambiae*; Am, *Apis mellifera*; Nv, *Nasonia vitripennis*; Bm, *Bombyx mori*; Ms, *Manduca sexta*; Ha, *Helicoverpa armigera*; Tn, *Trichoplusia ni*; Pr, *Pieris rapae*; AP, *Acyrthosiphon pisum*; Mp, *Myzus persicae*; Dn, *Diuraphis noxia*; Td, *Thermobia domestica*. *Td*AA15A and *Td*AA15B are underlined. **b** Phylogenetic tree of insect group I LPMO15s (LPMO15-1s). Amino acid sequences of LPMO15-1 proteins from different insect species were obtained by performing a BlastP search of NCBI database. *Tc*LPMO15-2 (group II LPMO15) of *T. castaneum* was used as the outgroup. Phylogenetic trees were constructed by MEGA 7.0 software using the Neighbor-Joining method. See Supplementary Table 3 for the accession numbers of LPMO15-1 proteins used in this study.

By our initial search using the *Td*AA15A of *T. domestica* as a query, we identified nine genes in the genome of *T. castaneum* that encode LPMO15 (AA15 family)-like proteins. Using these predicted LPMO protein sequences as queries, we searched other insect genomes that have been fully sequenced and annotated. A phylogenetic analysis of these sequences indicated that insect LPMO15-like proteins can be divided into at least four major clusters, which we denoted as groups I (LPMO15-1), II (LPMO15-2), III (LPMO15-3) and IV (lepidopteran-specific LPMO15) (Fig. 1a). Interestingly, 21 *Td*LPMO15s identified in the gut proteome of *T. domestica*^8^ comprise a separate clade. The groups *LPMO15-1* and *LPMO15-2* present in all orders of Insecta appear to have a single representative in all of the species identified, whereas the number of representatives in the *LPMO15-3* group ranged from one to seven in different species. Note that group III *LPMO15-3s* do not include any representatives from lepidopteran species characterized so far. Instead, there is a separate group with representatives from lepidopterans only, often with more than one member. All LPMO15-like proteins identified consist of a putative signal peptide and an AA15 catalytic domain. In addition, LPMO15-1s from group I have a C-terminal stretch containing a cysteine-rich motif, whereas LPMO15-2s from group II have a predicted transmembrane span.

### Identification and characterization of *TcLPMO15-1*, *LmLPMO15-1* and *OfLPMO15-1* cDNAs

For this study we focused only on members of the LPMO15-1 subgroup because their single-copy orthologous genes encoding not only a AA15 catalytic domain but also a C-terminal cysteine-rich motif are present in all insect orders so far examined, indicating that they are likely to have essential physiological functions. In addition to the insect genomes that have been fully sequenced and annotated, we also performed BLAST searches of the *L. migratoria* and *O. furnacalis* transcriptomes using the *Tc*LPMO15-1 and/or *Td*AA15A protein as queries. We identified LPMO15-1 orthologs from *L. migratoria* (*Lm*LPMO15-1) and *O. furnacalis* (*Of*LPMO15-1) as well as from species of other insect orders including the Coleoptera, Hymenoptera, Diptera, Hemiptera, Orthoptera, and Isoptera (Fig. 1b). Using primers flanking the predicted start and stop codon regions, we were able to amplify *TcLPMO15-1*, *LmLPMO15-1* and *OfLPMO15-1* cDNAs including the entire open-reading frames that encode proteins with 337, 339 and 343 amino acid residues and theoretical molecular masses for the mature proteins of 35.8, 34.6, and 35.3 kDa, respectively (Supplementary Fig. 1). Each protein, as seen in the members of group I LPMO15s, has a putative signal peptide and a conserved 192 or 193 amino acids-long AA15 catalytic domain followed by a ~120 amino acids-long C-terminal stretch containing a 74 amino acids-cysteine-rich motif that contains two 6-cysteine-containing internal repeats (C-X_15_-C-X_3_-C-X_6-9_-C-X_4_-C-X_1_-C) (Supplementary Fig. 2). In the AA15 catalytic domain of *Td*AA15A, two histidines (His1 and His91) directly coordinate a copper ion with a T-shaped geometry in the catalytic site, which is known as the histidine brace. Alanine (Ala89) and tyrosine (Tyr184) are also involved in forming the copper-containing active center as non-coordinating amino acid residues occupying the apical site and axial position of the copper ion, respectively. Two other tyrosines (Tyr24 and Tyr166) located at the boundaries of the flat surface surrounding the active center are involved in substrate binding^8^. All insect LPMO15-1-like proteins in our analysis (Supplementary Fig. 2) have the 192 amino acids-long AA15 catalytic domain except for some orthopteran LPMO15-1s, which are composed of 193 amino acids. All members of this group share a high degree of amino acid sequence identity/similarity (54–99% and 78–100%, respectively). The catalytically critical amino acid residues (e.g., H21, Y44, A109, H111, W188, and F206 in *Tc*LPMO15-1) occupy the same positions in the sequence alignment. All of them differ from the *Td*AA15A enzyme in the replacement of Y166 by a tryptophan (e.g., W188 in *Tc*LPMO15-1) in the boundary of the flat surface surrounding the active center and Y184 by a phenylalanine (e.g., F206 in *Tc*LPMO15-1) in the axial position of the copper-binding site (Supplementary Fig. 2).

### Developmental and tissue-specific expression of *LPMO15-1* genes in *T. castaneum* and *L. migratoria*

**Fig. 2:**
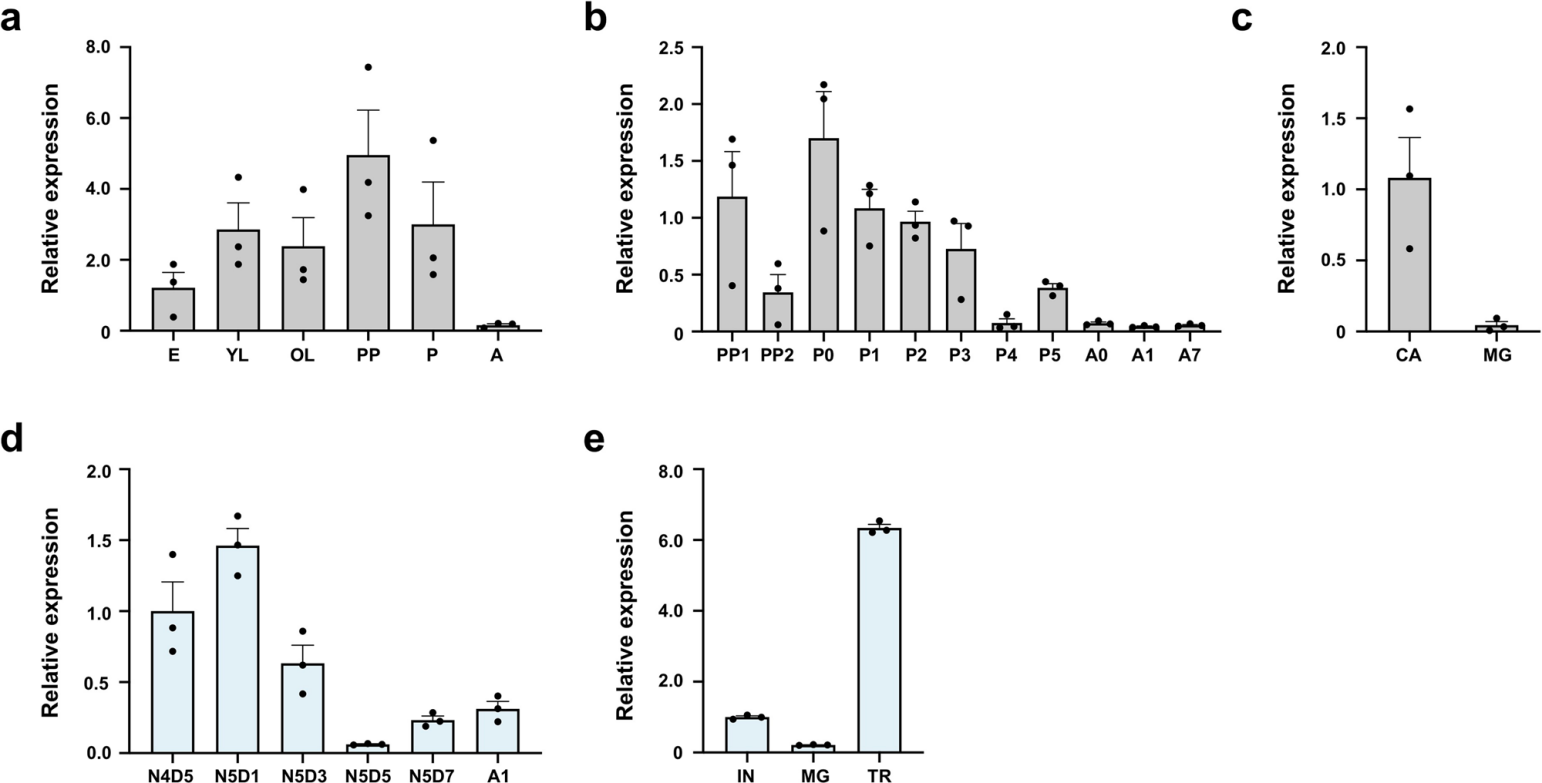
Expression profiles of *TcLPMO15-1* and *LmLPMO15-1*. **a** Transcript levels of *TcLPMO15-1* relative to that of *TcRpS6* at the indicated developmental stages of *T. castaneum* were determined by real-time qPCR. E embryos; YL young larvae; OL old larvae; PP pharate pupae; P pupae; A mature adults. **b** To analyze the expression patterns of *TcLPMO15-1* at later stages of development, the time-points analyzed were expanded between the early pharate pupa to young adult stage. PP1, day 0-1 pharate pupae; PP2, day 1–2 pharate pupae; P0, day 0 pupae; P1, day 1 pupae; P2, day 2 pupae; P3, day 3 pupae; P4, day 4 pupae; P5, day 5 pupae; A0, day 0 adults; A1, day 1 adults; A7, day 7 adults. **c** To analyze the transcript levels of *TcLPMO15-1* in the carcass (CA, whole body minus midgut) and midgut (MG), cDNA was prepared from total RNA extracted from a pool of tissues of ten actively feeding larvae. **d** Transcript levels of *LmLPMO15-1* relative to that of *LmRP49* in the integument collected from the indicated developmental stages of *L. migratoria* were determined by real-time qPCR. N4D5, 4th instar day 5 nymph; N5D1, 5th instar day 1 nymph; N5D3, 5th instar day 3 nymph; N5D5, 5th instar day 5 nymph; N5D7, 5th instar day 7 nymph; A1, adult day 1. **e** To analyze spatial expression patterns of *LmLPMO15-1*, total RNA was extracted from the integument (IN), midgut (MG) and trachea (TR) from three 5th instar day 3 nymphs. All data are shown as the mean value ± SE (*n* = 3).

To determine the role(s) of LPMO15-1s in insect development, the expression profiles of *TcLPMO15-1* and *LmLPMO15-1* during various growth stages were conducted using real-time PCR. With *T. castaneum*, transcripts of *TcLPMO15-1* were detected in all developmental stages analyzed with lowest expression in embryos and mature adults and highest expression in the pharate pupal stage (Fig. 2a). During late stages of development from pharate pupae to day 7 adults, high transcript levels of *TcLPMO15-1* were detected at early pharate pupal (PP1) and early pupal (P0-P2) stages, which declined thereafter (Fig. 2b). To assess the tissue specificity of expression of *TcLPMO15-1*, we dissected late-stage larvae to obtain midgut and carcass (whole body minus midgut). The transcript level of *TcLPMO15-1* in the carcass was substantially higher than that in the midgut (Fig. 2c). Similarly, high transcript level of group II *TcLPMO15-2* was also detected in the carcass but not in the midgut. Contradictorily, all seven group III *TcLPMO15-3s* were exclusively expressed in the midgut but not in the carcass (Supplementary Fig. 3), suggesting functional specialization of *TcLMPO15-3*s have potential important roles in digestion of dietary chitin and/or peritrophic matrix-associated chitin, but not in cuticular chitin turnover. Recently, Qu et al.^33^ reported that a midgut-specific group III *LmLPMO15-3*-like gene in *L. migratoria* is essential for the deconstruction of the peritrophic matrix during molting.

In *L. migratoria*, the temporal expression pattern of *LmLPMO15-1* was analyzed from the 4^th^ instar nymph to the adult stage. Transcripts of *LmLPMO15-1* were observed at all stages with higher levels detected in the 4th instar day 5 and 5th instar days 1–3 during the nymph-nymph molt (Fig. 2d). The tissue-specific expression analysis showed that *LmLPMO15-1* was expressed at the highest level in the trachea and then the integument, and the lowest level in the midgut (Fig. 2e). These results suggest a role of both *Tc*LPMO15-1 and *Lm*LPMO15-1 in the turnover of chitin in the cuticle and trachea.

### Biochemical properties of *Of*LPMO15-1

**Fig. 3:**
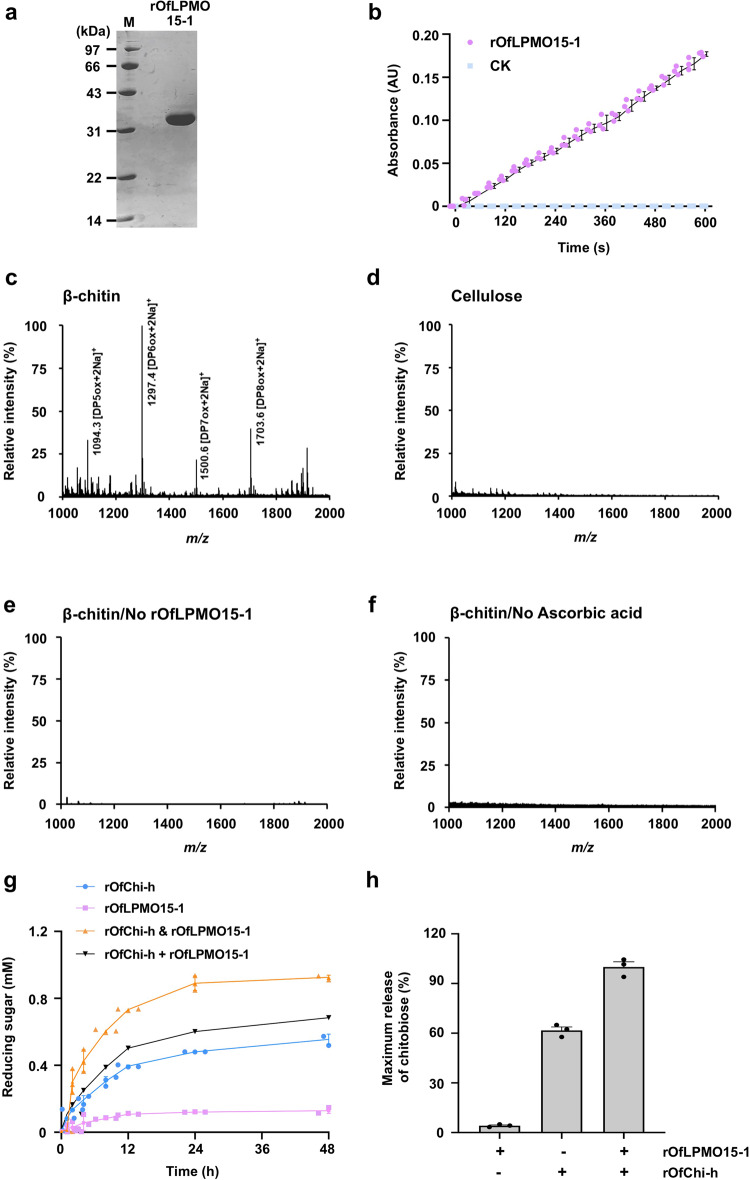
Enzymatic properties of r*Of*LPMO15-1. **a** SDS-PAGE analysis. r*Of*LPMO15-1 protein obtained by β-chitin bead affinity chromatography was subjected to electrophoresis on a 15% SDS-PAGE and stained with Coomassie Brilliant Blue R-250. **b** Enzymatic activity. r*Of*LPMO15-1 (1 μM) was incubated with 5 mM 2,6-DMP and 100 μM H_2_O_2_ in 100 mM sodium phosphate (pH 6.0) at 30 °C. The absorbance at 469 nm was measured every 30 s up to 600 s (red dots). The same assay without r*Of*LPMO15-1 was performed as a negative control (blue dots). **c**–**f** LPMO substrate specificity testing with β-chitin and cellulose. r*Of*LPMO15-1 (1 μM) was incubated with 2 mg/ml β-chitin (**c**) or microcrystalline cellulose (**d**) in 20 mM sodium phosphate buffer (pH 6.0) containing 1 mM ascorbic acid at 30 °C for 24 h followed by centrifugation at 17,000 × *g*. The supernatants were analyzed by MALDI-TOF/TOF mass spectrometry. The same assays with β-chitin without r*Of*LPMO15-1 (**e**) or ascorbic acid (**f**) were performed as negative controls. **g** The reducing sugar generated by the LPMO-chitinase system. Each reaction mixture contained 2 mg/ml β-chitin, 5 μM r*Of*LPMO15-1 and/or 1 μM r*Of*Chi-h in 20 mM sodium phosphate buffer (pH 6.0). An extra of 1 mM ascorbic acid was added in the presence of r*Of*LPMO15-1. “r*Of*LPMO15-1 + r*Of*Chi-h” indicates the calculated sum of the reducing sugar generated by the individual enzymes. The “r*Of*LPMO15-1 & r*Of*Chi-h” indicates the reducing sugar produced by combining these two enzymes in the reaction. **h** Relative product quantification showing the synergistic effect between r*Of*LPMO15-1 and r*Of*Chi-h. Each reaction mixture contained 2 mg/ml β-chitin, 1 mM ascorbic acid, 5 μM r*Of*LPMO15-1 and/or 1 μM r*Of*Chi-h in 20 mM sodium phosphate buffer (pH 6.0). The soluble products generated were quantified by HPLC. The soluble products generated by r*Of*LPMO15-1 alone was first degraded by r*Of*Chi-h and then quantified by HPLC. Chitobiose was the main product generated from chitin degradation, so that the synergistic effect was calculated according to the amount of chitobiose in each sample. All data are shown as the mean value ± SE (*n* = 3).

To further illustrate that LPMO15-1s participate in the degradation of cuticular chitin during molting, we attempted to express the full-length proteins, *Tc*LPMO15-1, *Lm*LPMO15-1 and *Of*LPMO15-1 from *T. castaneum*, *L. migratoria* and *O. furnacalis*, respectively, in yeast cells. Only the recombinant expression of *Of*LPMO15-1, which shares an amino acid sequence identity of 71% and 63% with those of *Tc*LPMO15-1 and *Lm*LPMO15-1, respectively, was successful (Fig. 3a). The recombinant *Of*LPMO15-1 was found to have a peroxidase activity when using 2,6-dimethoxyphenol (2,6-DMP) as a chromogenic substrate and H_2_O_2_ as a co-substrate at pH 6.0 and 30 °C (Fig. 3b). The reaction was linear over a 10-min reaction time and the specific activity of the purified enzyme was estimated to be about 16.0 U/g under the experimental conditions, comparable to the values obtained for two enzymes purified from *C. gestroi* (6.2 and 7.6 U/g)^20^. r*Of*LPMO15-1 also oxidatively cleaved β-chitin and produced a series of chitin oligosaccharides in aldonic acid or lactone forms. The mass-to-charge ratio (*m/z*) profiles of the digestion products in the mass spectral analysis consisted of oxidized oligosaccharides with different degrees of polymerization (DP). The products corresponding to even-numbered oligosaccharides with DP6 and DP8 having higher intensities than the odd-numbered products with DP5 and DP7 (Fig. 3c). In addition, the peak masses of the adduct of the sodium salt of the oxidized chitooligosaccharide products showed a predominant C1-oxidation pattern. No oxidized product was detected either in the enzyme- or ascorbic acid-omitted negative controls (Fig. 3e, f) as well as in a reaction where micro-cellulose was used as the substrate (Fig. 3d), suggesting LPMO15-1s are not involved in cellulose degradation.

A potential synergistic effect between r*Of*LPMO15-1 and recombinant chitinase h (r*Of*Chi-h, an exo-acting processive chitinase in lepidopteran species) was examined using β-chitin as the substrate. As shown in Fig. 3g, a mixture of r*Of*LPMO15-1 and r*Of*Chi-h generated more reducing sugar from the substrate than the sum generated from reactions catalyzed by individual enzymes, indicating a total degradative potential of the binary LPMO15-1-chitinase system. To verify the potential synergistic effect between r*Of*LPMO15-1 and r*Of*Chi-h, HPLC analysis was then utilized to quantify the sugar products produced by the reactions (Fig. 3h). The production of reducing sugar and chitobiose obtained from using the binary enzyme cocktail was substantially greater than those obtained from combining the production obtained when utilizing the individual enzymes separately. The results suggest a synergistic effect between r*Of*LPMO15-1 and r*Of*Chi-h in chitin degradation.

### Effect of RNAi for *TcLPMO15-1* and *LmLPMO15-1* on insect molting and survival

Double-stranded RNA (dsRNA)-mediated transcript down-regulation was performed to determine the roles of *TcLPMO15-1* and *LmLPMO15-1* in development and molting of *T. castaneum* and *L. migratoria*, respectively. To analyze the transcript abundance for those *LPMO15-1* genes after RNAi, real-time qPCR experiments were carried out. Injection of dsRNA for *TcLPMO15-1* (ds*TcLPMO15-1*) into the last instar larvae led to a substantial depletion of *TcLPMO15-1* transcripts at the young pupal stage (day 0 pupae) (Supplementary Fig. 4a) when the targeted gene is maximally expressed (see Fig. 2b). Similarly, injection of ds*LmLPMO15-1* into the 5^th^ instar day 1 nymphs caused a substantial decrease in the level of *LmLPMO15-1* transcripts (Supplementary Fig. 4b). In both species, injection of dsRNA for *LPMO15-1* had no significant effect on the transcript level of *LPMO15-2* that, like *LPMO15-1*, is predominantly expressed in cuticle-forming tissues (Supplementary Fig. 4).

**Fig. 4:**
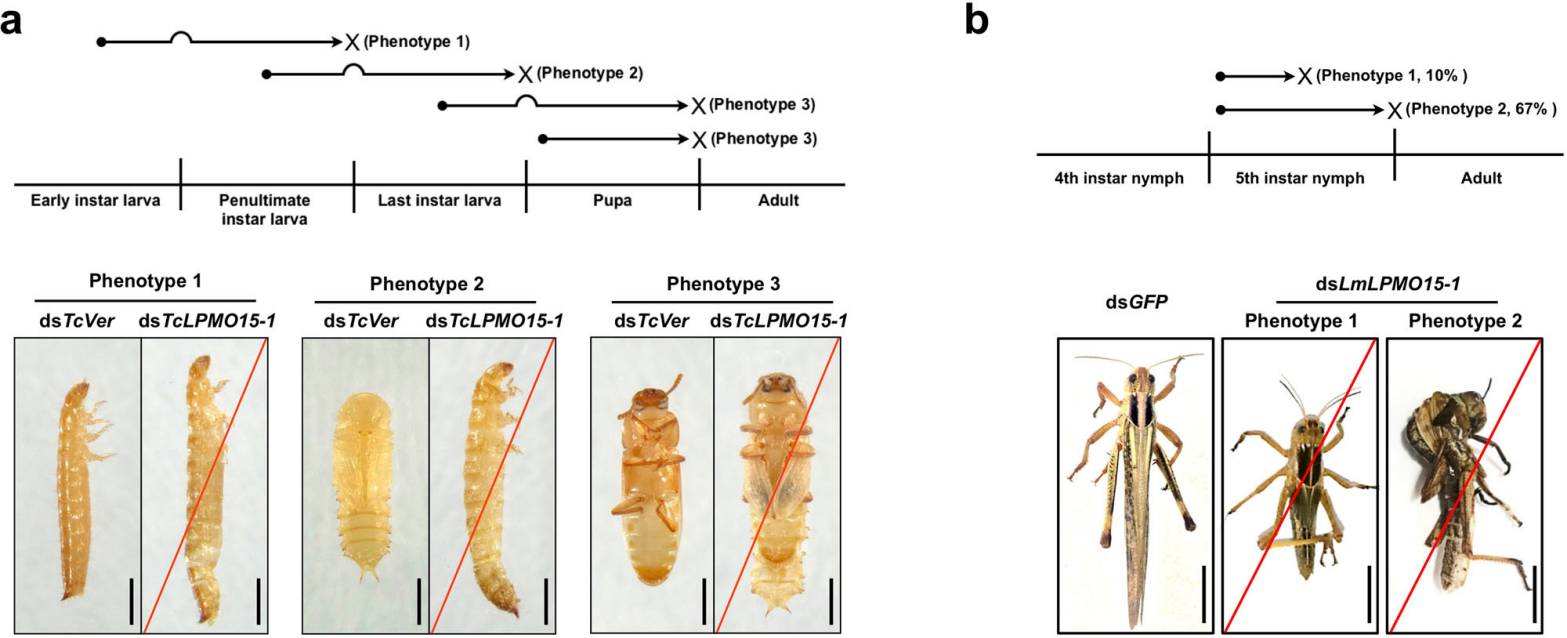
Lethal phenotypes produced by RNAi for *LPMO15-1* in *T. castaneum* and *L. migratoria*. **a** In *T. castaneum*, injection of ds*TcLPMO15-1* (200 ng per insect) into early instar, penultimate and last instar larvae had no effect on their subsequent molts and the resulting insects developed normally. However, they failed to undergo penultimate-last instar larval (Phenotype 1, *n* = 120), larval-pupal (Phenotype 2, *n* = 120) and pupal-adult (Phenotype 3, *n* = 120) molts, respectively, and died entrapped in their exuviae (red lines). A similar pupal-adult molting defect (*n* = 60) was also obtained when ds*TcLPMO15-1* was injected into day 0 pupae. Scale bar = 1 mm. **b** In *L. migratoria*, injection of ds*LmLPMO15-1* (10 µg per insect; injected twice) into 5th instar day 1 (first injection) and day 3 (second injection) nymphs resulted in developmental arrest and death (red lines) without dorsal splitting (Phenotype 1, ∼10%, *n* = 3) or exhibiting a dorsal splitting during the molt but entrapped in their nymphal cuticle (Phenotype 2, ∼70%, *n* = 20). Scale bar = 1 cm.

In *T. castaneum*, injection of ds*TcLPMO15-1* into early instar larvae had no effect on the subsequent molt and the resulting penultimate instar larvae developed normally (Fig. 4a). However, all of the larvae failed to complete the molt to the last larval instar (phenotype 1 in Fig. 4a). Slippage of the old larval cuticle was observed, but the penultimate instar larvae were trapped inside the old cuticle and died. When penultimate instar larvae were treated with ds*TcLPMO15-1*, the insects molted normally to the last instar larvae, but they subsequently failed to complete the larval-pupal molt and died as pharate pupae entrapped in the old larval cuticle (Phenotype 2 in Fig. 4a). When ds*TcLPMO15-1* was injected into last instar larvae, developmental arrest occurred during the pupal-adult molt when the pharate adults became entrapped in their pupal cuticle. Pupal cuticle slippage was evident, but the insects were unable to shed the pupal exuvium and died (Phenotype 3 in Fig. 4a). Similarly, injection of ds*TcLPMO15-1* into day 0 pupae also caused an incomplete pupal-adult molting in all of the insects (Phenotype 3 in Fig. 4a). These results indicate that *TcLPMO15-1* is required for all types of molting (larval-larval, larval-pupal and pupal-adult) in *T. castaneum*.

In contrast, injection of ds*LmLPMO15-1* into the 5^th^ instar day 1 nymphs resulted in terminal developmental arrest. Approximately 10% of the nymphs died without dorsal splitting (Phenotype 1 in Fig. 4b), while ~70% of the nymphs initiated a molting process exhibiting both a dorsal split and new adult cuticle. However, the insects failed to shed the antecedent cuticle and died (Phenotype 2 in Fig. 4b). All of these results suggest that insect LPMO15-1 appears to play an essential role during the molting process, presumably in turnover of cuticular chitin, which is critical for completion of the molt. To evaluate this hypothesis further, we analyzed by TEM the ultrastructure of cuticles from *TcLPMO15-1*- and *LmLPMO15-1*-deficient insects.

### Ultrastructure of cuticles from LPMO15-1-deficient insects

**Fig. 5:**
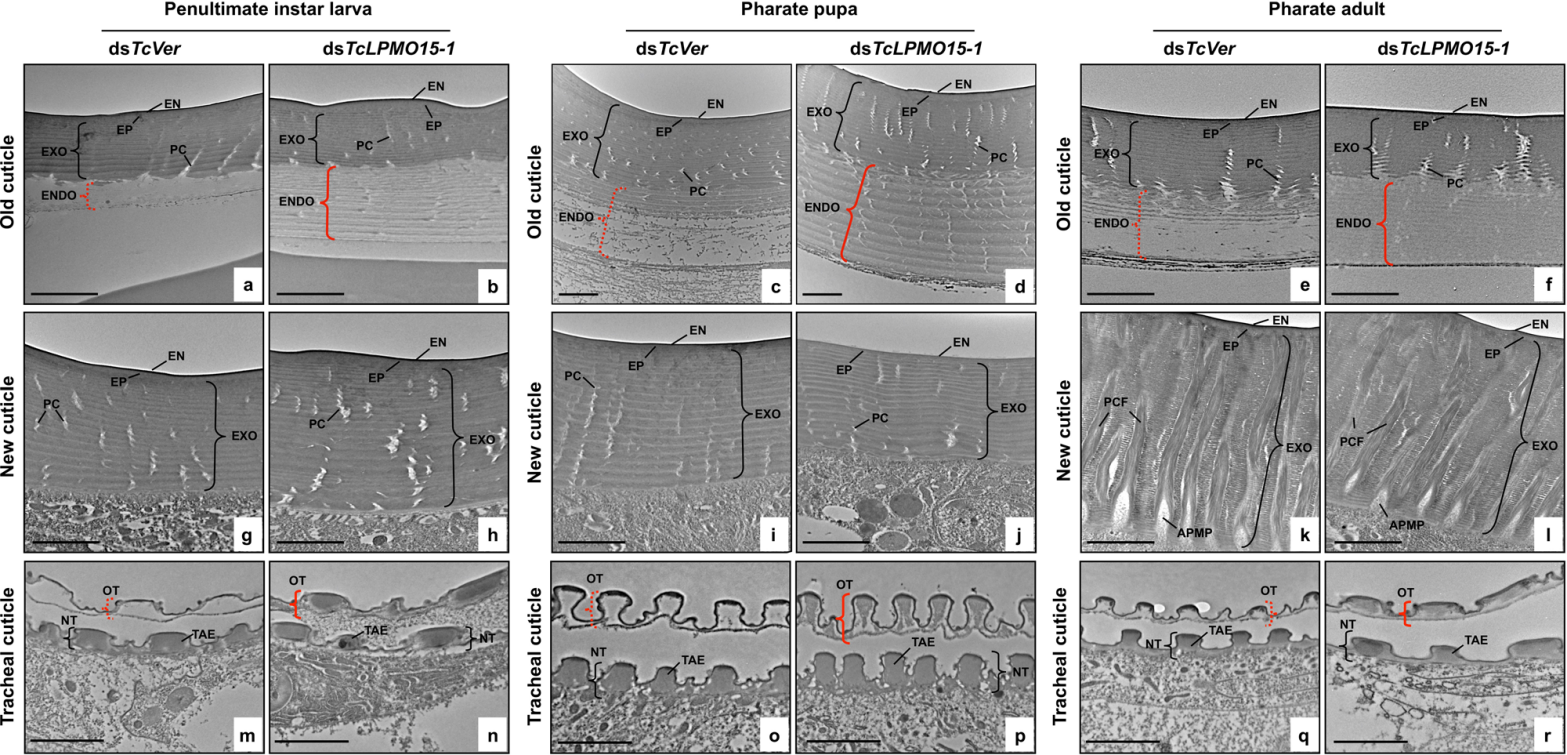
Ultrastructure of cuticles from *Tc*LPMO15-1-deficient larvae, pharate pupae, and pharate adults of *T. castaneum*. Ultrastructure of old (**a**–**f**), newly forming cuticles (**g**–**l**), and tracheal cuticle (**m**–**r**) from penultimate instar larvae, pharate pupae, and pharate adults that had been injected with ds*TcLPMO15-1* or ds*TcVer* at early instar, penultimate, and last instar larval stages, respectively, was analyzed by TEM. ds*TcVer*-treated controls showed degradation of the endocuticle (ENDO) in the overlying old cuticle (red dotted brackets) during each molt analyzed, while those of *Tc*LPMO15-1-deficient insects were intact, retaining a number of chitinous horizontal laminae (red solid brackets). Similarly, ds*TcVer*-treated controls exhibited degradation of the old tracheal cuticle (red dotted brackets), while the old tracheal cuticle of *Tc*LPMO15-1-deficient insects was incompletely degraded and contained electron-dense materials (red solid brackets). EN, envelope; EP, epicuticle; EXO, exocuticle; ENDO, endocuticle; PC, pore canal; PCF, pore canal fiber; APMP, apical plasma membrane protrusion. OT, old tracheal cuticle; NT, new tracheal cuticle; TAE, taenidia. Scale bar = 2 µm.

As loss of function of *LPMO15-1* by RNAi caused molting arrests in both *T. castaneum* and *L. migratoria*, we analyzed by TEM the morphology and ultrastructure of the old and newly forming cuticles during molting periods in both species. In *T. castaneum*, there were no obvious differences in morphology and thickness (Supplementary Table 1) of the newly forming body wall cuticles of ds*TcLPMO15-1*-treated insects isolated at the penultimate larval instar, pharate pupal and pharate adult stages when compared with the ds*TcVer*-treated controls. New cuticles from both control and *Tc*LPMO15-1-depleted insects exhibited well-organized, horizontal alternating electron-dense and electron-lucent chitinous laminae, as well as vertically oriented helicoidal pore canals in the larval and pupal cuticles in addition to wide vertical structures with a central chitin fiber core of pore canal fibers (PCFs) in the adult cuticle (Fig. 5g–l). In the old cuticles at each molting stage analyzed, the endocuticles of ds*TcVer*-treated controls had been degraded (Fig. 5a, c, e). In contrast, RNAi for *TcLPMO15-1* caused failure of turnover of the endocuticular layer in which the horizontal chitinous laminae and vertical pore canals remained essentially intact (Fig. 5b, d, f), resulting in a significantly thicker cuticle compared to that of the ds*TcVer*-controls (Supplementary Table 1).

As a high transcript level of *LPMO15-1* was detected in the tracheae of several insect species^8,33^, we analyzed the ultrastructure of tracheal cuticle in *Tc*LPMO15-1-depleted *T. castaneum*. Like that seen in the external cuticles, the old tracheal cuticles of ds*TcVer*-treated controls had been degraded (Fig. 5m, o, q), whereas those from ds*TcLPMO15-1*-treated insects were incompletely digested and remained electron-dense (Fig. 5n, p, r). There was no obvious difference in morphology of the new tracheal cuticles including the taenidial fold arrangement between ds*TcVer*-controls and ds*TcLPMO15-1*-animals (Fig. 5m–r).

**Fig. 6:**
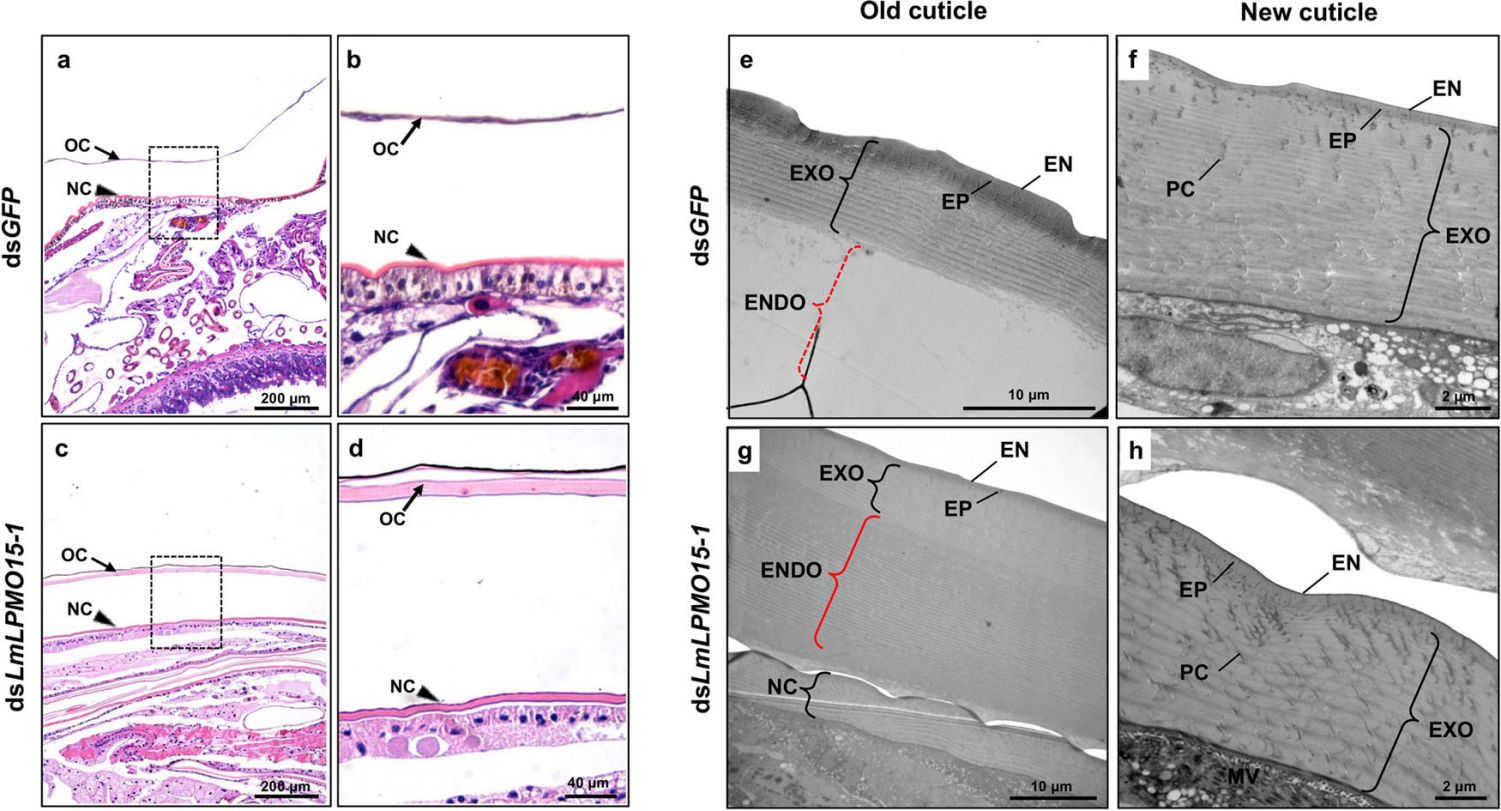
Ultrastructure of cuticle from pharate adult of *Lm*LPMO15-1-deficient *L. migratoria*. **a**–**d** Paraffin sections (5 μm) of third abdominal segments from pharate adults (5th instar day 9 nymphs) that had been injected with ds*LmLPMO15-1* or ds*GFP* as the 5th instar day 1 nymphs were stained with hematoxylin and eosin. The old nymphal cuticle (OC) and newly forming adult cuticle (NC) (box in **a**, **c**) were enlarged in **b**, **d**. The OC of ds*LmLPMO15-1*-insects was significantly thicker than that of ds*GFP*-controls. **e**–**h** Ultrastructure of the old and new cuticles from each dsRNA-treated insect were analyzed by TEM. ds*GFP*-treated controls showed the degraded endocuticle (ENDO) in the overlying old cuticle (red dotted bracket in **e**), while that of ds*LmLPMO15-1*-treated insects remained intact, exhibiting numerous chitinous horizontal laminae (red solid bracket in **g**). EN, envelope; EP, epicuticle; EXO, exocuticle; ENDO, endocuticle; PC, pore canal.

To investigate the functional importance of *Lm*LPMO15-1 in turnover/morphology of the old and/or new cuticles during adult eclosion in *L. migratoria*, those tissues were dissected from the third abdominal segment of pharate adults (5^th^ instar day 9 nymphs) that had been injected with ds*GFP* (control) or ds*LmLPMO15-1* on 5^th^ instar day 1 and again on day 3. Paraffin sections of tissues stained with hematoxylin and eosin showed that the old cuticle of ds*LmLPMO15-1*-treated insects was much thicker than that of ds*GFP*-treated controls (Fig. 6a–d and Supplementary Table 1), suggesting that *Lm*LPMO15-1-deficient insects had failed to digest the old cuticle. To confirm that the turnover of the old cuticle was affected, we further performed TEM analysis of old and new cuticles. The endocuticular portion of the old cuticle of ds*GFP*-control insects was nearly completely digested, whereas that of ds*LmLPMO15-1*-treated insects appeared to be intact, retaining numerous horizontal chitin laminae and vertical pore canals (Fig. 6e, g). As observed with cuticles of *Tc*LPMO15-1-depleted *T. castaneum*, RNAi for *LmLPMO15-1* yielded no obvious differences in thickness and morphology of either the horizontal laminae or vertical pore canals in the newly formed adult cuticle compared with that of the ds*GFP*-control (Fig. 6f, h and Supplementary Table 1). All of these results indicate that LPMO15-1 plays a role during molting in digestion of chitin in the old cuticle, which is critical for the completion of the molt, but not in the formation of new cuticle or its morphology including the chitinous laminar organization and pore canal/PCF structure in both *T. castaneum* and *L. migratoria*.

## Discussion

The degradation of extracellular matrix polysaccharides in insects and plants presents special challenges because of the crystallinity and possible cross-linking of the substrates. A commonly held view envisaged that a combination of families of endo- and exo-acting hydrolytic enzymes could accomplish the digestion of recalcitrant polysaccharides including chitin and cellulose^34,35^. However, the relatively recent discovery that proteins collectively known as LPMOs, which bind to these substrates, promote utilization of insoluble substrates and have oxidase activities of their own, has provided new insight about the mechanism of turnover of naturally occurring polysaccharides by microbes as well as arthropods^8,13–17,27–29^. LPMOs from microbes break the C-H bond of either C1 or C4 carbons of polysaccharides, resulting in aldonic acid or lactone products. This process results in the generation of internal entry sites in the polysaccharide for the hydrolytic enzymes such as chitinases and cellulases in addition to facilitating the decrystallization of chitin or cellulose chains from crystalline bundles^36^.

Besides microbes, arthropods also contain a variety of LPMO-like proteins. Recently, analysis of the gut proteome and transcriptome of the ancient insect species, *T. domestica* from the order Zygentoma, has confirmed the expression in the gut of a large family of LPMO15s belonging to the AA15 family of enzymes. These proteins promote digestion of dietary crystalline cellulose without the assistance of gut microbes, unlike termite species that require such microbial assistance for cellulose digestion. Individual *Td*LPMO15s differed in their ability to act on either chitin or cellulose or both. On the other hand, two LPMO15s from *C. gestroi* could act only on chitin and not on cellulose, indicating their role in structural matrix remodeling rather than in food digestion. So far, the only evidence for the idea that LPMO15s might have a role in insect cuticle turnover comes from genome-wide RNAi of *D. melanogaster* and the iBeetle project that identified pupal lethality and other defects in *T. castaneum* (http://ibeetle-base.uni-goettingen.de/). However, there have been no direct studies on chitin turnover in the cuticle or on the enzymatic activity/specificity of LPMO15-like proteins in these species. Our study addresses these specific issues and points to an essential role for these enzymes in cuticular chitin turnover, which is not met alone by the assortment of endo- and exochitinases.

There is only one representative from each insect species in LPMO15 groups I and II, which are distantly related phylogenetically. The LPMO15-1 subgroup is of an ancient origin and must have diverged from a common progenitor that gave rise to the LPMO15-2 subgroup. For instance, LPMO15-like genes (AA15 family genes) have been found in many invertebrate species including marine animals such as mollusks and many arthropods such as chelicerates, arachnids, myriapods, and crustaceans^8^. In addition, the highly conserved group I LPMO15-1-like proteins appear to be present in not only insect species but also crustaceans including *Daphnia pulex* (EFX74851, EFX74850), *D. magna* (XP_032794104, XP_032793818), *Penaeus japonicas* (XP_042868838, XP_042868839), *Procambarus clarkii* (XP_045584551), and *Hyalella azteca* (XP_018007939). Results from these searches suggest that group I LPMO15-1s are the founding members of the AA15 family in pancrustaceans originally coming from the sea and then colonizing the land where they might play key roles in cuticle turnover (more discussion below), and that the less conserved midgut-specific group III LPMO15-3s developed afterwards for specialized roles in digestion in response to different diets and/or in degradation of PM-associated chitin.

LPMO15 groups I and II have distinctly different protein domain distributions. All group I LPMO15-1s have two copies of a 6-cysteine repeat often associated with chitin-binding domains. However, these cysteine repeats are not related to the CBM14 domain present in insect chitinase-like proteins^37^. Group II LPMO15-2s lack this domain but have a predicted C-terminal transmembrane domain. Group III LPMO15-3 proteins in several insect genomes contain redundant enzymes that have only the AA15 catalytic domain. Group IV proteins have an interesting distribution among insects, being prevalent only in lepidopteran species. The significance of these unique distributions remains unknown.

The presence of these distinct groups among insect species suggests functional differences among them. This inference is further supported by the limited data that we currently have on tissue specificity of expression of these LPMO15 groups. Groups I LPMO15-1 enzymes from both *T. castaneum* and *L. migratoria* are expressed predominantly in cuticular-enriched tissues including the integument and trachea, but not in the midgut. These tissue-specific expression profiles of *LPMO15-1* are consistent with those of genes encoding group I (CHT5/ChtI) and group II (CHT10/ChtII) chitinases and β-*N*-acetylglucosaminidase 1 (NAG1), all of which are known to play a role in cuticular chitin degradation, as well as the expression of gene encoding chitin synthase-A (CHS-A), which catalyzes the synthesis of cuticular chitin in both species (Supplementary Fig. 5)^38–43^, suggesting their involvement in chitin metabolism in the cuticle rather than in a midgut digestive function. They are also expressed at comparable periods in the molting cycle and not in the adult stage.

Besides exhibiting peroxidase activity with 2,6-DMP as the substrate, the highly purified recombinant *Of*LPMO15-1 oxidized crystalline α- and β-chitins, but not cellulose, yielding chitooligosaccharide products consistent with a role in chitin turnover, but not in the digestion of dietary cellulose, similar to *Td*AA15B of *T. domestica*. It should be emphasized that besides sharing a high degree of amino acid sequence identity, the three LPMO15-1 proteins from *T. castaneum, L. migratoria* and *O*. *furnacalis* have the same four essential amino acids (H21/30/32, H111/120/122, A109/118/120 and F206/216/217, respectively) involved in the histidine brace structure containing the catalytic Cu atom first identified in the *Td*AA15B enzyme, which is specific for chitin^8^.

Finally, we demonstrated by RNAi that the LPMO15-1 enzymes in two different insect orders are critical for insect molting as well as cuticle digestion. Administration of dsRNA for the single copy of the gene encoding this enzyme resulted in molting failure in *T. castaneum* at all developmental stages. In cases other than the pupal stage, the insects managed to molt to the next developmental stage (larva, prepupa, or pupa) but died without completing the next molt cycle due to a high level of transcript depletion of the targeted gene. While we do not have a precise explanation for the delay in the development of the phenotype until the next molt cycle, we hypothesize a delay in LPMO protein depletion due to a long half-life of the protein past the point of transcript depletion.

This report is the first demonstration of the essential role of an LPMO in promoting the turnover of the chitinous cuticle. The failure to molt after depletion of a single oxidative enzyme supports the hypothesis that LPMOs are as equally important as the chitinolytic enzymes in the digestion of cuticle chitin at each molt cycle. The turnover of chitin in the old cuticle by molting fluid chitinases presents several challenges because of the crystallinity of cuticular chitin. Thus, LPMO15-1 apparently acts to oxidatively cleave microcrystalline chitin chains, which facilitates the delamination/decrystallization of chitin to generate new substrate binding sites for chitinolytic enzymes. The insect binary LPMO-chitinase system acts synergistically to efficiently digest chitin in the old cuticle during the molting process. In a comparable microbial polysaccharide-degrading system, Nguyen et al.^44^ recently demonstrated that an LPMO (*Cs*LPMO9) from the fungus, *Ceriporiopsis subvermispora*, together with a commercial cellulase cocktail cooperated synergistically to depolymerize microcrystalline cellulose.

For practical applications in economic and medical entomology, LPMOs should be evaluated as a potential target for manipulating insect pest populations. Further study of the critical roles of LPMOs in insect growth and development via comparative and functional insect genomics may facilitate the development of a novel anti-LPMO insect control strategy.

## Methods

### Insects

The GA-1 strain of *T. castaneum*^45^ was used for this study. Beetles were reared at 30 °C and 50% relative humidity in whole wheat flour containing 5% brewer’s yeast^46^. *L. migratoria* was kindly provided by the Institute of Zoology, Chinese Academy of Sciences (CAS). Nymphs were reared on fresh wheat sprouts in the laboratory at 28 °C under a 14 h light/10 h dark diurnal cycle.

### Cloning of *TcLPMO15-1*, *LmLPMO15-1*, and *OfLPMO15-1* cDNAs

*T. castaneum*, *L. migratoria*, and *O. furnacalis* homologs of *Td*AA15A from *T. domestica* (accession number: GASN01405718.1) were identified by performing a BLAST search of the *T. castaneum* genome and the *L. migratoria* and *O. furnacalis* transcriptomes. To clone *TcLPMO15-1* cDNA, total RNA was isolated from a pool of six whole *T. castaneum* pupae (mixture of day 0–5 pupae) by using the RNeasy Mini Kit (Qiagen, Valencia, CA, USA). First-strand cDNAs were synthesized with the Super Script III First-Strand Synthesis System (Invitrogen, Carlsbad, CA, USA) using an oligo-(dT)_18_ primer. To clone *LmLPMO15-1* and *OfLPMO15-1* cDNAs, total RNA was isolated from *L. migratoria* 5^th^ instar day 1 nymphs and *O. furnacalis* pharate pupae, respectively, using Trizol reagent (Invitrogen), and then treated with DNase I (Takara, Dalian, China). First-strand cDNAs were synthesized using the Reverse Transcriptase M-MLV (Takara) and used as template for cDNA amplification. cDNAs containing the predicted full-length coding sequence of *TcLPMO15-1*, *LmLPMO15-1* and *OfLPMO15-1* were amplified by PCR using the gene-specific primers shown in Supplementary Table 2. The cDNA fragments were cloned into pGEM-T (Promega, Madison, WI, USA) or pEASY-T1 vector (TransGen Biotech, Beijing, China) and sequenced. GenBank accession numbers of the *TcLPMO15-1*, *LmLPMO15-1* and *OfLPMO15-1* clones are MZ636451, MZ440879 and MZ440880, respectively.

### Protein sequence and phylogenetic analysis

LPMO-like proteins in fully sequenced or well annotated insect genomes/transcriptomes were identified by a BLAST search of the NCBI database using the *Td*AA15A protein sequence as the query. Multiple sequence alignment of proteins was carried out using the ClustalW software tool (https://www.genome.jp/tools-bin/clustalw). SignalP-5.0 (http://www.cbs.dtu.dk/services/SignalP/) was used to predict signal peptides. LPMO domains were identified using the Conserved Domain Database (CDD, https://www.ncbi.nlm.nih.gov/cdd). A core region of homologous sequence highly conserved in all LPMO15 proteins analyzed, including a AA15 catalytic domain, was aligned. A phylogenetic tree was constructed with the MEGA 7 program^47^ using the neighbor-joining method. See Supplementary Table 3 for the accession numbers of LPMO15-1 proteins used for amino acid sequence alignment and phylogenetic analysis.

### Gene expression analysis by real-time qPCR

To analyze temporal and spatial expression patterns of *TcLPMO15-1* and *LmLPMO15-1*, total RNA was isolated from embryos, young larvae, old larvae, pharate pupae, pupae and adults, larval midgut and carcass (whole body without midgut) of *T. castaneum*; and integument of 4th instar day 5, 5th instar day 1, 3, 5, and 7 and adult day 1; and midgut and tracheal tissues dissected from 5th instar day 3 of *L. migratoria*. For *TcLPMO15-1*, real-time PCR was done in a 40 μl reaction volume containing 1 μl of template cDNA, 20 μl TB Green Premix Ex Taq (Takara), 0.25 μM of each primer using the Thermal Cycler Dice real-time PCR system III (Takara). For *LmLPMO15-1*, real-time qPCR was performed in a 20 µl reaction volume containing 10 µl TransStart Top Green qPCR SuperMix (TransGen Biotech), 2 µl template cDNA and 0.2 µM of each primer using the Real-time PCR Detection System LightCycler480II (Roche, Indianapolis, USA). Transcript levels of the *T. castaneum* ribosomal protein S6 (*TcRpS6*) or *L. migratoria* ribosomal protein 49 (*LmRp49*) were measured to normalize for differences among the concentrations of cDNA templates. Each sample included three biological replicates and three technical replicates. The relative expression levels for each gene were calculated relative to the reference gene according to the 2^−ΔΔCt^ method^48^. See Supplementary Table 4 for the primer sequences used for real-time qPCR experiments.

### Expression of recombinant *Of*LPMO15-1 protein

The coding sequence excluding the putative signal peptide of *Of*LPMO15-1 was optimized to the yeast codon bias for yeast expression, synthesized the DNA template (Taihe Biotechnology, Beijing, China) and used for PCR amplification using the primer set: 5´-AGA AGG GGT ATC TCT CGA GAA AAG ACA TGG AAG ATT GAT GGA CCC-3´ and 5´-GAA TTA ATT CGC GGC CGC TTA GTA ACA CCT ACA CCT GT-3´. The forward and reverse primers contain *Xho I* and *Not I* recognition sites (underlined), respectively, to facilitate directional cloning into the pPIC9 vector (Invitrogen). The PCR product was digested with *Xho I* and *Not I* and subcloned into the same sites of the pPIC9 plasmid DNA behind the signal cleavage site with α-factor at the N-terminus in frame. The recombinant plasmid was linearized using *Sac I* and then transformed into *Pichia pastoris* strain GS115. Positive clones were selected and the recombinant *Of*LPMO15-1 protein (r*Of*LPMO15-1) was obtained by induction with 1% methanol for 120 h. r*Of*LPMO15-1 was purified by 75% saturation of ammonium sulfate precipitation, followed by affinity chromatography using β-chitin beads^49^. The precipitate was resuspended in distilled water and centrifuged at 17,000 × *g* for 30 min at 4 °C. The supernatant was then passed through a 0.2 μm filter and desalted in buffer A (20 mM Tris-HCl, pH 8.0) containing 500 mM NaCl and 0.05% TrionX-100. Afterward, the sample was applied on β-chitin beads equilibrated with buffer A. After incubation at 4 °C for 1 h, the β-chitin beads were washed three times with 10 volumes of buffer A. The bound protein was eluted by adding 2 volumes of 20 mM acetic acid and then neutralized by adding sodium phosphate (pH 6.0) to a final concentration of 100 mM. The purity of affinity-purified r*Of*LPMO15-1 was analyzed by 15% SDS-PAGE.

### Enzyme assay

Enzymatic activity of the purified r*Of*LPMO15-1 was measured by utilizing the peroxidase activity associated with this class of enzymes using 2, 6-dimethoxyphenol (2,6-DMP) and H_2_O_2_ as co-substrates^50^ with modifications. The reaction solution containing 5 mM 2,6-DMP and 100 µM H_2_O_2_ in 200 µl of 100 mM sodium phosphate (pH 6.0) was preincubated at 30 °C for 10 min, and then the absorbance at 469 nm was measured every 30 s shortly after adding r*Of*LPMO15-1 (final concentration of 5 µM). In the control group, the reaction solution contained the same components except that no enzyme was added. For determination of activity of r*Of*LPMO15-1 toward chitin and cellulose, r*Of*LPMO15-1 (5 µM) was incubated with 2 mg/ml β-chitin or microcrystalline cellulose (Sigma-Aldrich, St. Louis, MO, USA) in 300 µl of 20 mM sodium phosphate buffer (pH 6.0) containing 1 mM ascorbic acid at 30 °C for 24 h with rotation. In the control group, the reaction solution contained the same components except that no enzyme or ascorbic acid was added. The reaction mixture was centrifuged at 17,000 × *g* for 10 min and 0.5 µl of the reaction product in the supernatant was analyzed by matrix-assisted laser desorption/ionization-time of flight (MALDI-TOF) mass spectrometry performed on the MALDI micro MX system (Waters, Milford, MA, USA). Processive glycoside hydrolases are potential enzymes to utilize for testing whether there is synergy between LPMOs and glycoside hydrolases. *Of*Chi-h is an exo-acting processive chitinase that exhibits high activity toward crystalline chitin. It was recombinantly expressed using the *P. pastoris* expression system and purified from the cell culture supernatant using metal-chelating chromatography as previously described^35^. β-chitin was used to detect the degradative potential of the LPMO-chitinase system. For the single enzyme hydrolysis condition, a final concentration of 1 µM r*Of*Chi-h or 5 µM r*Of*LPMO15-1 was added in a total volume of 1.0 ml containing 100 mM sodium phosphate buffer (pH 6.0) and 2 mg/ml chitin in a 2 ml Eppendorf tube. For the two-enzyme combination condition, 1 μM r*Of*Chi-h and 5 μM r*Of*LPMO15-1 were added to the reaction system together. In addition, 1 mM ascorbic acid was added to the reaction mixture whenever LPMO was included in the assay. These tubes were incubated horizontally in an incubator at 200 rpm for 48 h at 30 °C. A 0.06 ml sample was withdrawn from well-mixed digestion mixtures at selected time-points during digestions. An aliquot of 0.18 ml potassium ferriferrocyanide (2 mg/ml) was then added and the mixture was boiled for 15 min. The amount of reducing sugar generated corresponded with the potassium ferriferrocyanide consumption, which was quantified by measuring the absorbance at 420 nm. All of the assays were performed in triplicate. To quantify a possible synergistic effect between r*Of*LPMO15-1 and r*Of*Chi-h, a final concentration of 5 µM r*Of*LPMO15-1 and/or 1 µM r*Of*Chi-h was added in a total volume of 0.1 ml containing 100 mM sodium phosphate buffer (pH 6.0), 1 mM ascorbic acid and 2 mg/ml β-chitin in a 1.5 ml Eppendorf tube. All the assays were performed in triplicate. These tubes were incubated in an incubator at 200 rpm for 24 h at 30 °C. Four hundred microliters of ethanol was added to stop the reaction. The soluble products generated by r*Of*LPMO15-1 alone were further degraded by 1 µM r*Of*Chi-h for another 2 h before ethanol was added. Then, the supernatants of all samples were collected after centrifugation and dried down. The resulting pellets were resuspended in 100 μl of pure water and the soluble sugars were then analyzed using a TSK-Gel Amide-80 column (0.46 × 25 cm) (Tosoh, Tokyo, Japan) on a HPLC system (Agilent Technologies, Santa Clara, USA) as previously described^51^. The HPLC analysis indicated that chitobiose was the main product generated from chitin degradation, such that the synergistic effect was calculated according to the amount of chitobiose in each sample.

### RNA interference (RNAi)

Templates for synthesis of double-stranded RNA (dsRNA) for *TcLPMO15-1* (ds*TcLPMO15-1*) and *LmLPMO15-1* (ds*LmLPMO15-1*) were amplified by PCR using the gene-specific primers containing T7 RNA promoter sequences at the 5′-ends. ds*TcLPMO15-1* and ds*LmLPMO15-1* were synthesized using an AmpliScribe T7-Flash kit (Epicenter Technologies, Madison, WI, USA) and T7 RiboMAX™ Express RNAi System (Promega, Madison, WI, USA), respectively^38,52^. In *T. castaneum*, ds*TcLPMO15-1* (200 ng per insect) was injected into early instar larvae, penultimate instar larvae, last instar larvae or day 0 pupae (*n* = 20–40 for each of the three independent experiments). To analyze knockdown levels of *TcLPMO15-1* transcripts, total RNA was isolated from whole day 0 pupae (*n* = 3) that had been injected with dsRNA at last instar larval stage. In *L. migratoria*, ds*LmLPMO15-1* (10 µg per insect) was injected into the 5th instar day 1 nymphs (1st injection), and the same amount of dsRNA was injected a second time 3 days after the first injection to increase RNAi efficiency (*n* = 10 for each of the three independent experiments). To analyze knockdown levels of *LmLPMO15-1* transcripts, total RNA was isolated from the integument of 5th instar day 5 nymphs (2 days after 2nd dsRNA injection). dsRNAs for *T. castaneum Vermilion* (ds*TcVer*), encoding tryptophan oxygenase, was used as both a negative control for non-specific effects of dsRNA and as a positive control for monitoring effectiveness of RNAi by monitoring the eye pigmentation (the *TcVer* gene affects only eye-color) of *T. castaneum*^53^. dsRNAs for green fluorescent protein (ds*GFP*) was synthesized^52^ and injected to serve as a negative control for *L. migratoria*. The primer sequences used and lengths of the dsRNAs are listed in Supplementary Table 2.

### Histochemistry

*L. migratoria* pharate adults (5th instar day 9 nymphs) that had been injected with ds*LmLPMO15-1* or ds*GFP* into 5th instar day 1 nymphs were collected, and their third abdominal segments were dissected. Samples were fixed in 4% paraformaldehyde for 24 h, dehydrated in an ethanol gradient of 30, 50, 70, 90, and 100% for 30 min each, and then embedded in paraffin^43^. Paraffin sections (5 μm) were stained with hematoxylin and eosin (Beyotime Biotechnology, Haimen, China) and then observed using the Olympus IX-83 inverted microscope.

### Transmission electron microscopy (TEM)

Ultrastructure of cuticles from the dsRNA-treated animals was analyzed by TEM^54^. Penultimate instar larvae, pharate pupae (day 2 prepupae) and pharate adults (day 5 pupae) of *T. castaneum* that had been treated previously with ds*TcLPMO15-1* or ds*TcVer* were collected and fixed in a mixture of 4% paraformaldehyde and 0.1% glutaraldehyde in 0.1 M sodium cacodylate buffer (pH 7.4) for 24 h at room temperature. Samples were rinsed three times for 15 min with 0.1 M sodium cacodylate buffer, and then dehydrated in an ethanol gradient of 50, 60, 70, 80, 90, 95, and 100% for 20 min each. The tissues were infiltrated in LR white resin (Electron Microscopy Sciences, PA, USA) in 2:1 ethanol: resin for 4 h, 1:1 ethanol: resin for 8–10 h, 1:2 ethanol: resin for 4 h and 100% resin for 4 h. Tissues were vacuum-infiltrated for 2 h, placed in gelatin capsules (Electron Microscopy Sciences), and then polymerized at 55 °C for 12–16 h followed by ultrathin sectioning. Ultrathin sections (~90 nm) were stained with 4% aqueous uranyl acetate for 10 min and then imaged using the JEM-1400 transmission electron microscope (JEOL Ltd. Tokyo, Japan). In the case of samples of *L. migratoria*, pharate adults (5th instar day 9 nymphs) that had been injected with ds*LmLPMO15-1* or ds*GFP* at the 5th instar day 1 nymph stage were collected, and the integument of their third abdominal segments were dissected and fixed in a mixture of 4% paraformaldehyde and 0.1% glutaraldehyde in 0.1 M phosphate buffer (pH 7.4) for 24 h at room temperature. Samples were washed with 0.1 M phosphate buffer, and then fixed with 1% osmium tetroxide for 3 h. The tissues were rinsed with phosphate buffer, dehydrated in acetone, and then embedded in Epon 812 (Sigma-Aldrich, St. Louis, MO, USA) for 2 h at room temperature and baked in a 62 °C oven for 48 h, followed by ultrathin sectioning. Ultrathin sections were stained with 4% aqueous uranyl acetate for 10 min and then imaged using the JEM-1200EX transmission electron microscope (JEOL Ltd. Tokyo, Japan).

### Statistics and reproducibility

Data are presented as mean ± standard error (SEM) from at least three independent replicates. The Student *t*-test was used to analyze for a statistical difference between two samples (pairwise comparison). The specific number of replicates (and individuals) and the statistically significant (*p*-value) in each experiment are summarized in the corresponding Methods section and/or figure legends. The graphs were generated using GraphPad Prism 9 (https://www.graphpad.com/scientific-software/prism/).

### Reporting summary

Further information on research design is available in the Nature Research Reporting Summary linked to this article.

## Supplementary information

Supplementary Information

Suplementary data 1

Reporting Summary

Description of Additional Supplementary Files

## Data Availability

All related data are available within the manuscript or in Supplementary Data 1. Accession numbers for gene sequences analyzed and their sources can be found in Supplementary Tables 2.
