# Supplementary material for: AA15 lytic polysaccharide monooxygenase is required for efficient chitinous cuticle turnover during insect molting
Source: Commun Biol. 2022 May 31;5:518. doi: 10.1038/s42003-022-03469-8 (PMC9156745; doi:10.1038/s42003-022-03469-8)
Supplement: Supplementary file 1 — Supplementary Information [file 42003_2022_3469_MOESM1_ESM.pdf]

Supplementary Table 1. Thickness of old and newly forming cuticles from LPMO15-1-deficient *T. castaneum* and *L. migratoria*.

Species	Developmental stage analyzed	Cuticles	Thickness of cuticle (μm)		*p* value
			ds*TcVer*	ds*TcLPMO15-1*	
T. castaneum	Penultimate instar larvae	old larval cuticle	1.86 ± 0.09	4.50 ± 0.28	1.89E-07
		new larval cuticle	3.30 ± 0.12	4.00 ± 0.48	0.18
	Pharate pupae	old larval cuticle	1.51 ± 0.12	6.05 ± 0.33	1.66E-13
		new pupal cuticle	3.09 ± 0.21	3.23 ± 0.11	0.54
	Pharate adult	old pupal cuticle	3.36 ± 0.32	7.27 ± 0.14	4.52E-09
		new adult cuticle	7.10 ± 0.25	7.03 ± 0.17	0.81

Species	Developmental stage analyzed	Cuticles	Thickness of cuticle (μm)		*p* value
			ds*GFP*	ds*LmLPMO15-1*	
L. migratoria	Pharate adult	old nymphal cuticle	14.66 ± 0.22	29.86 ± 0.77	3.67E-15
		new adult cuticle	8.79 ± 0.16	8.46 ± 0.34	0.39

Data are shown as the mean value ± SE (n = 9-15).

Supplementary Table 2. Primers used for cloning and dsRNA synthesis.

Gene	Genbank Accession #	Direction*	Sequence (5'-3')**	Product size (bp)	Note
TcLPMO15-1	MZ636451	F	ATGCTAGTGCCATTATCACG	1,014	cloning
		R	TTAATTCGTTGAAAATTCTTCG		
		F	(T7)-CAACGGCAAATGCGGCCT	295	dsRNA
		R	(T7)-GGCTTTCTTCTTTCCGTCT		
TcVer	AY052390	F	(T7)-CTGAGCGAAGAATGTGGGAT	603	dsRNA
		R	(T7)-GGTTAAAAGCTTCTCGACCG		
LmLPMO15-1	MZ440879	F	GGAGCAGCGGCGACACCACG	1,704	cloning
		R	CGCAAATTAAGCTGTTCAGC		
		F	(T7)-AAGTTGAGGTGGACCTGACG	413	dsRNA
		R	(T7)-TGCGGAAGTCTCGGTAGTAGA		
OfLPMO15-1	MZ440880	F	TCGCGCCATTCAAAACTTCG	2,120	cloning
		R	poly(T)		
GFP	CVU76561	F	(T7)-GCACCATCTTCTTCAAGGA	231	dsRNA
		R	(T7)-GATGTTGTGGCGGATCTT		

*F, forward; R, reverse.
**T7, T7 RNA polymerase recognition sequence (TAATACGACTCACTATAGGG).

Supplementary Table 3. Accession numbers of insect LMPO15-1s used for the amino acid sequence alignment and phylogenetic analysis.

Order	Name	Species	Accession number
Coleoptera	*At*LPMO15-1	*Aethina tumida*	XP_019876627
	*Agl*LPMO15-1	*Anoplophora glabripennis*	XP_018574059
	*Dp*LPMO15-1	*Dendroctonus ponderosae*	XP_019763018
	*Ld*LPMO15-1	*Leptinotarsa decemlineata*	XP_023029101
	*Nve*LPMO15-1	*Nicrophorus vespilloides*	XP_017786569
	*Ot*LPMO15-1	*Onthophagus taurus*	XP_022909937
	*Tc*LPMO15-1	*Tribolium castaneum*	MZ636451
Lepidopetra	*Bm*LPMO15-1	*Bombyx mori*	XP_004926014
	*Ha*LPMO15-1	*Helicoverpa armigera*	XP_021180988
	*Ms*LPMO15-1	*Manduca sexta*	XP_030035714
	*Of*LPMO15-1	*Ostrinia furnacalis*	MZ440880
	*Px*LPMO15-1	*Papilio xuthus*	XP_013180709
	*Pr*LPMO15-1	*Pieris rapae*	XP_022121259
	*Tn*LPMO15-1	*Trichoplusia ni*	XP_026747704
Hymenoptera	*Am*LPMO15-1	*Apis mellifera*	XP_026298239
	*Nv*LPMO15-1	*Nasonia vitripennis*	XP_001602613
Diptera	*Aa*LPMO15-1	*Aedes aegypti*	XP_021696109
	*Aal*LPMO15-1	*Aedes albopictus*	XP_019525791
	*Ag*LPMO15-1	*Anopheles gambiae str. PEST*	XP_310852
	*Cc*LPMO15-1	*Ceratitis capitata*	XP_012157461
	*Dm*LPMO15-1	*Drosophila melanogaster*	NP_001188545
	*Hi*LPMO15-1	*Hermetia illucens*	CAD7090791
	*Md*LPMO15-1	*Musca domestica*	XP_019893458
Orthoptera	*Asp*LPMO15-1	*Atractomorpha sp*	GDYX01023215
	*Lm*LPMO15-1	*Locusta migratoria*	MZ440879
	*Sg*LPMO15-1	*Schistocerca gregaria*	GHHP01008411
	*Sp*LPMO15-1	*Schistocerca piceifrons*	GIOR01128883
Hemiptera	*Ap*LPMO15-1	*Acyrthosiphon pisum*	XP_016660633
	*Ago*LPMO15-1	*Aphis gossypii*	XP_027852856
	*Al*LPMO15-1	*Apolygus lucorum*	KAF6203625
	*Cl*LPMO15-1	*Cimex lectularius*	XP_014240666
	*Dn*LPMO15-1	*Diuraphis noxia*	XP_015368218
	*Mp*LPMO15-1	*Myzus persicae*	XP_022170584
	*Nl*LPMO15-1	*Nilaparvata lugens*	XP_039292167
	*Rm*LPMO15-1	*Rhopalosiphum maidis*	XP_026816188
Isoptera	*Cs*LPMO15-1	*Cryptotermes secundus*	XP_023723964
	*Zn*LPMO15-1	*Zootermopsis nevadensis*	XP_021922611
Zygentoma	*Td*LPMO15-1	*Thermobia domestica*	GASN01008505

Supplementary Table 4. Primers used for real-time qPCR analysis.

Gene	Genbank Accession #	Direction*	Sequence (5'-3')	Product size (bp)
TcLPMO15-1	MZ636451	F	GACCCACATCACGTCAAAGA	91
		R	CTTGGCCGACGCTGTAAT	
TcLPMO15-2	XM_015980870	F	GGCGTTCCTCTTCGTCTTT	105
		R	TGAGGCGTATCGAAACCATATC	
TcLPMO15-3s	XM_015981790	F	ACGGAAGAAGAGGACAATGATAC	95
		R	GCAGTGTAGGTCCATCTCAAA	
	XM_015981792	F	CAACGTCTGGAAGACAGACTAC	99
		R	CCAGTTCGGTAATTCCACCTAA	
	XM_015981791	F	GAAGCTTGCAAACGGTGATAAA	119
		R	GATAATGCCAACGCAAGACAC	
	XM_963550	F	GCTTCCAGATGGGTTGACTTG	98
		R	CTTCTTGGTAACTCCCGTCTTC	
	XM_963476	F	AAATGGCGAGCCGAGATAC	100
		R	GACACATCTGTCGCACTTCA	
	XM_008201743	F	GTGCTCATTGTGTGTTGAGATG	94
		R	TCTTGAGACCCACATCCTTTG	
	XM_970659	F	TTGCTGATGGTTCCTCGAAATA	106
		R	CACCTCAAGACACACCTATCA	
TcCHT5	AY675073	F	AAATGGGACGAACACGGCAAAGTC	120
		R	CCCACCGTAGCCCTTTGATTTGAT	
TcCHT10	DQ659250	F	ATTGGTCAAGGCGTTGAAACTGGG	104
		R	AAGTCTTCAAGAGCGGGTGTTTGC	
TcNAG1	EF592536	F	CCGATAGTGCGAGTGTTGAT	98
		R	CTCGGCTTCTCTCCATGTATTC	
TcCHS-A	AY291475	F	GGTGGTCGATCCGGATTATTACGA	97
		R	GTTGACTTGCGTTTCGTCGTCGTT	
TcCHS-B	AY291477	F	TGCAGTGGTTTCGAAAGGGACAAC	154
		R	AATGAGGAGCCAGATCCATTGCAC	
TcRpS6	NM_001172390	F	ACGCAAGTCAGTTAGAGGGTGCAT	81
		R	TCCTGTTCGCCTTTACGCACGATA	
LmLPMO15-1	MZ440879	F	CTACTTCATCCCGCCAGGTA	80
		R	GGGTGCACGTCACGAAAGG	
LmLPMO15-2	GEZB01012450	F	ACACTTGTTGCGGTCTGTGC	116
		R	CGTGTCCTCGGAGGTGGAAC	
LmLPMO15-3	OK181108	F	TTCCGCACCTGCTCAGACAT	90
		R	CACGTTGTAGTCCGCGTTGG	
LmChtI	KM397371	F	CATCAAAGCGAAGGGCTACGGC	92
		R	AGATTAGTGCGTCCTTCGGGCCA	
LmChtII	KU757433	F	CTTTCAAACCACGAGGCTTACTC	158
		R	ATGACCAGTCTGCTTATCCCAAT	
LmNAG1	JX888720	F	CCCTCGCAGAACGTTTGTG	133
		R	TGTAGACACCACTCAGGCTCGAT	
LmCHS-A	GU067730	F	GCACTGTTCGTTCTCATCGTATTC	121
		R	ACCAAACCAATTGGCTCAAG	
LmCHS-B	JQ901491	F	CAGCCTTCCGCATAGACAACT	150
		R	CGGCCATCATAACCAATGAAT	
LmRp49	GETS01057474	F	CGTAAACCGAAGGGAATTGA	115
		R	GAAGAAACTGCATGGGCAAT	

*F, forward; R, reverse.

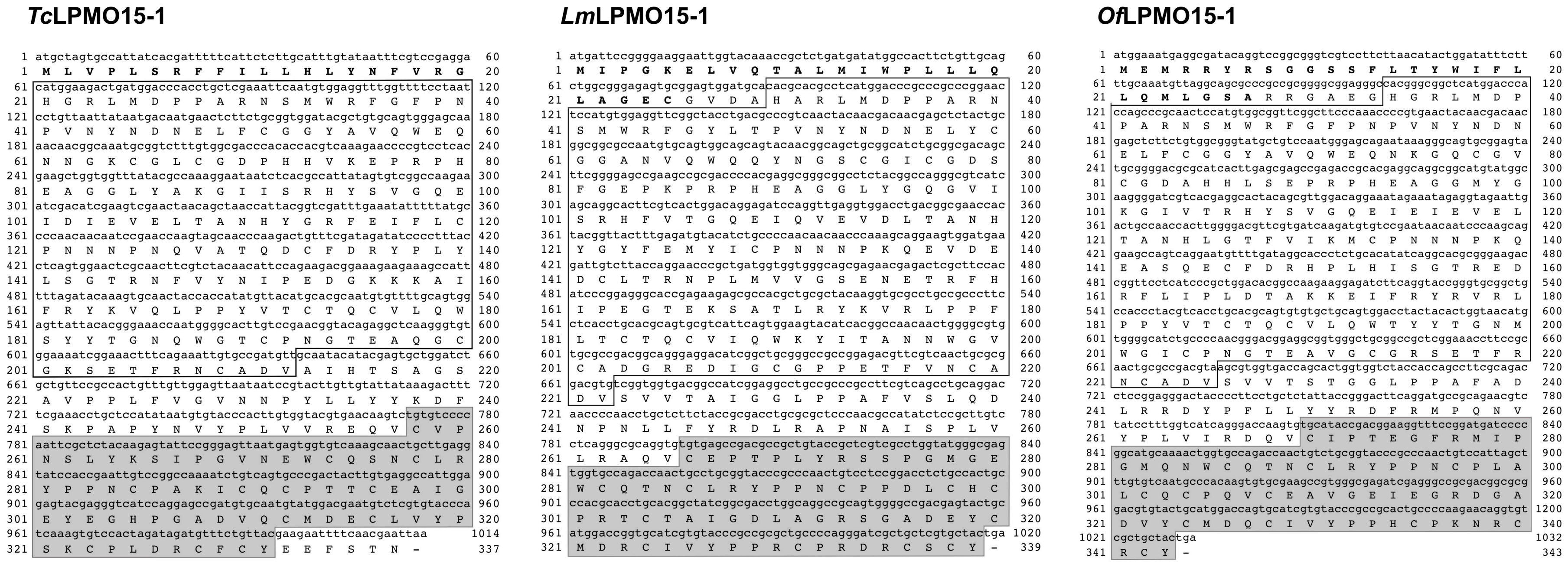

Supplementary **Fig. 1. Nucleotide and deduced amino acid sequences of *Tc*LPMO15-1, *Lm*LPMO15-1 and *Of*LPMO15-1.** Predicted secretion signal peptide sequences and the putative LPMO catalytic domains are bolded and boxed, respectively. The conserved cysteine-rich motifs identified at the C-terminus of insect LPMO15-1 sequences is indicated by gray highlight.

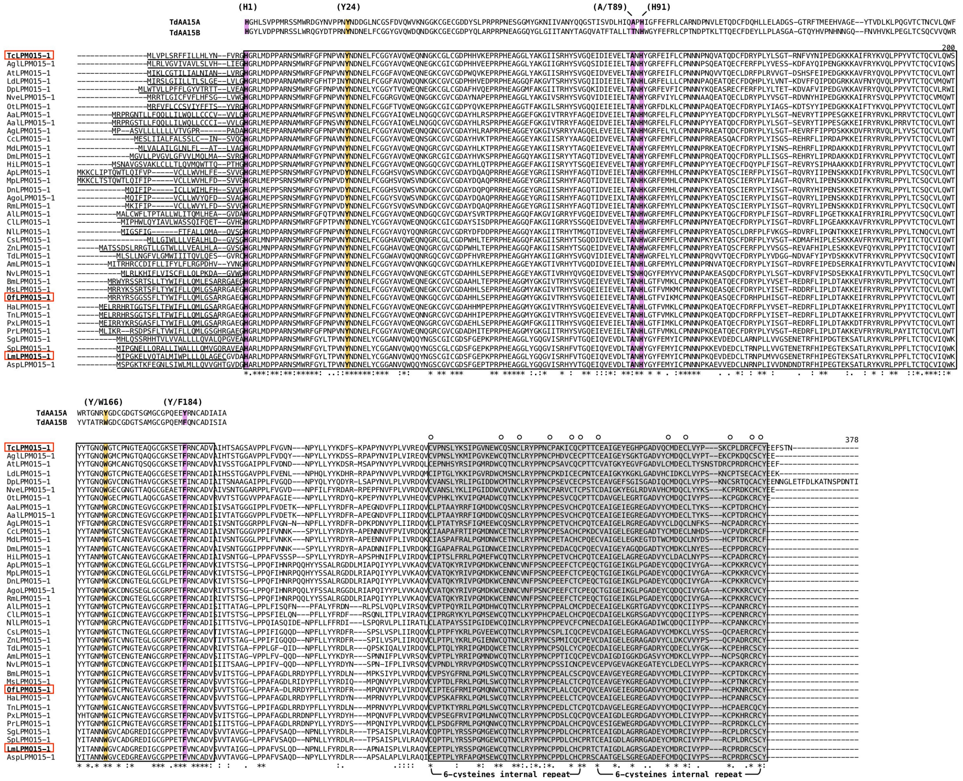

Supplementary Fig. 2. Amino acid sequence alignments of insect LPMO15-1s. Multiple protein sequence alignment of LPMO15-1s from several lepidopteran, coleopteran, hymenopteran, dipteran, hemipteran and orthopteran species was made using ClustalW software. Symbols located under the alignment indicate identical (*), highly conserved (:), and conserved residues (.) respectively. Amino acid sequences of the catalytic domains of *Td*AA15A and *Td*AA15B are also shown above the alignment. Predicted signal peptides and the putative LPMO15 catalytic domains are underlined and boxed, respectively. The four conserved amino acids (corresponding to H1, H91, A/T89 and Y/F184 in *Td*AA15A and *Td*AA15B) involved in the copper binding and the two (corresponding to Y24 and Y/W166 in *Td*AA15A and *Td*AA15B) involved in the substrate binding are highlighted in magenta and yellow, respectively. Gray highlight indicates C-terminal stretches consisting of two "6-cysteines-containing internal repeats ($C-X_{15}-C-X_3-C-X_{6-9}-C-X_4-C-X_1-C$)" where X is any amino acid residue. The 6-cysteines in each repeat are indicated by open circles.

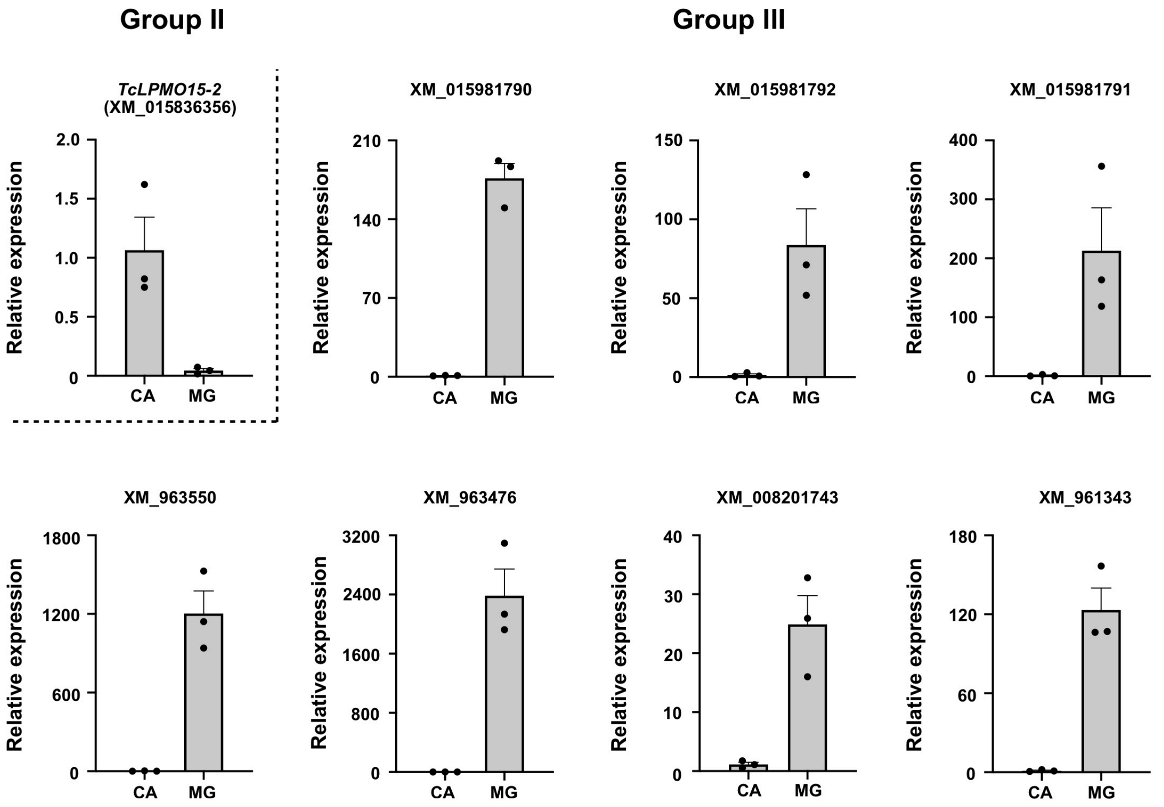

Supplementary Fig. 3. Tissue-specific expression of group II and group III LPMOs in *T. castaneum* by RT-qPCR. Transcript abundance of *TcLPMO15-2* (group II) and seven *TcLPMO15-3* (group III) genes in the carcass (CA) and midgut (MG) was determined by real-time qPCR. Total RNA was extracted from the tissues of *T. castaneum* larvae (n = 10). Expression levels of *TcLPMO15*s are presented relative to the levels of expression in carcass (CA). Data are shown as the mean values ± SE (n = 3)

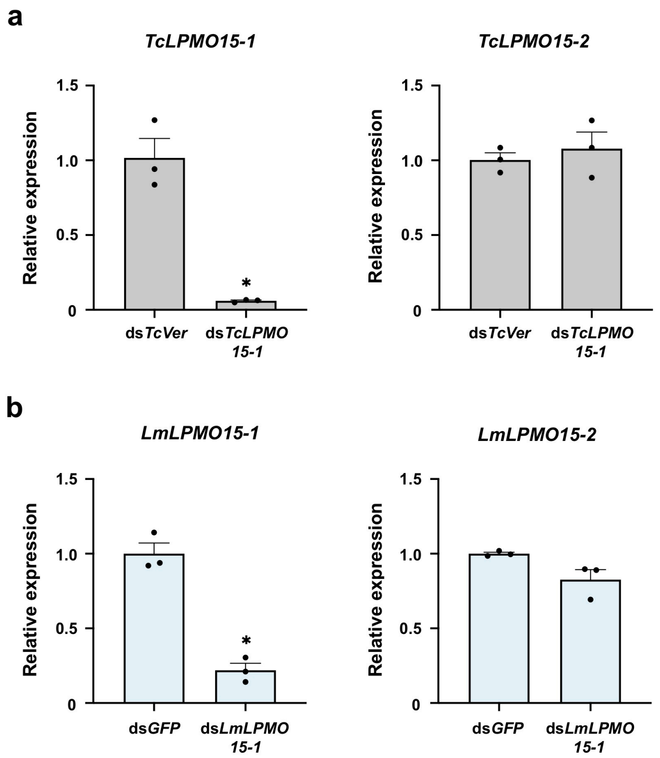

Supplementary Fig. 4. Knockdown levels of transcripts of *TcLPMO15-1* and *LmLPMO15-1* genes by RT-qPCR. a cDNAs were prepared from total RNA isolated from three pooled day 0 pupae that had been injected with ds*TcLPMO15-1* at the last instar larval stage. Transcript levels of *TcLPMO15-1* and *TcLPMO15-2* in ds*TcLPMO15*-1-treated insects were presented relative to the levels in ds*TcVer*-treated controls. An asterisk indicates a significant difference in transcript levels of *TcLPMO15-1* (p = 1.83E-03, t-test) between samples. Data are shown as the mean value ± SE (n = 3). **b** cDNAs were prepared from total RNA isolated from three pooled integument of 5th instar day 5 nymphs 2 d after the second ds*LmLPMO15-1* injection. Expression levels of *LmLPMO15-1* and *LmLPMO15-2* in ds*LmLPMO15-1*-treated insects were presented relative to the levels in ds*GFP*-treated controls. An asterisk indicates a significant difference in transcript levels of *LmLPMO15-1* (p = 7.93E-04, t-test) between samples. Data are shown as the mean values ± SE (n = 3).

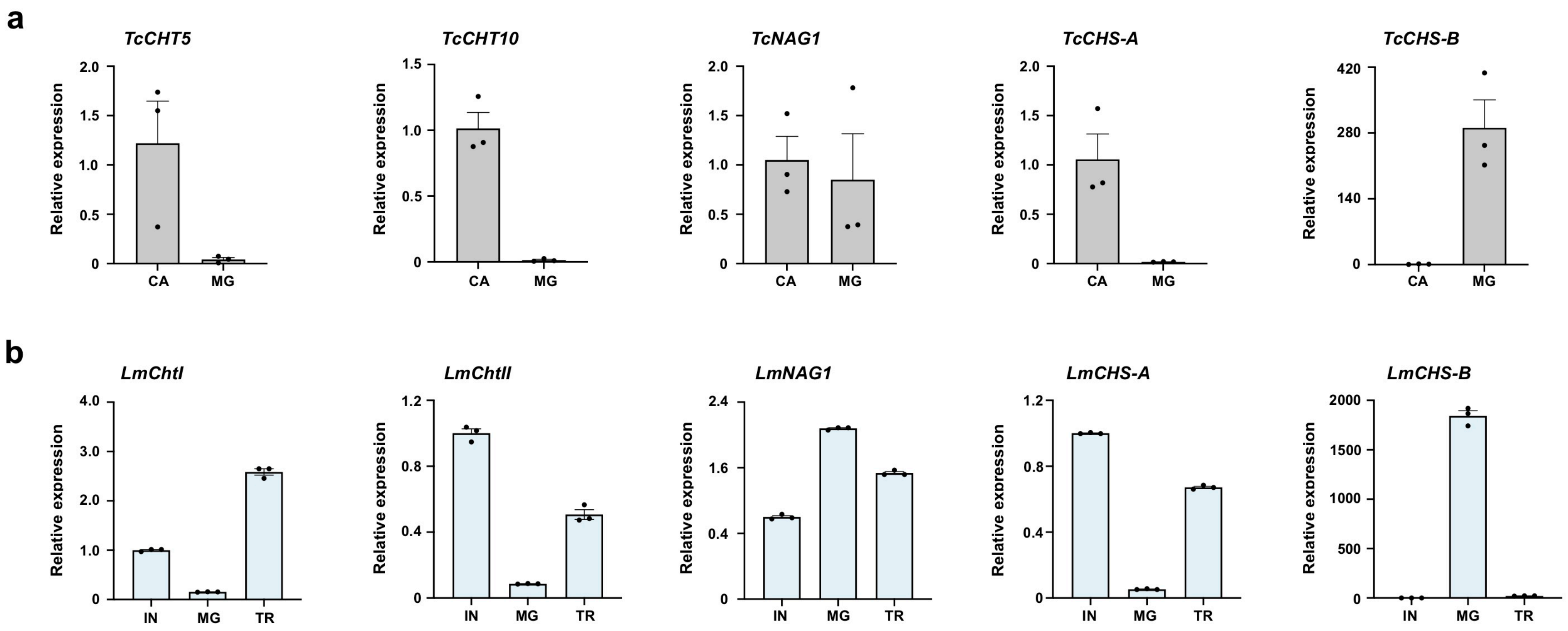

Supplementary Fig. 5 Tissue-specific expression of chitinases and chitin synthases in *T. castaneum* and *L. migratoria* by RT-qPCR. a Transcript abundance of *TcCHT5* (group I chitinase, AY675073), *TcCHT10* (group II chitinase, DQ659250), *TcNAG1* (β-*N*-acetylhexosaminidase, EF592536), *TcCHS-A* (chitin synthase-A, AY291475) and *TcCHS-B* (chitin synthase-B, AY291477) in the carcass (CA) and midgut (MG) was determined by real-time qPCR. Total RNA was extracted from the tissues of *T. castaneum* larvae (n = 10). Expression levels of each gene are presented relative to the levels of expression in carcass (CA). **b** To analyze spatial expression patterns of *LmCHTI* (group I chitinase, KM397371), *LmCHTII* (group II chitinase, KU757433), *LmNAG1* (JX888720), *LmCHS-A* (GU067730) and *LmCHS-B* (JQ901491), total RNA was extracted from the integument (IN), midgut (MG) and trachea (TR) from three *L. migratoria* 5th instar day 3 nymphs. Expression levels of each gene are presented relative to the levels of expression in integument (IN). All data are shown as the mean value ± SE (n = 3). See Supplementary Table 4 for the primer sequences used in this study.
