# Supplementary material for: AA15 lytic polysaccharide monooxygenase is required for efficient chitinous cuticle turnover during insect molting
Source: Commun Biol. 2022 May 31;5:518. doi: 10.1038/s42003-022-03469-8 (PMC9156745; doi:10.1038/s42003-022-03469-8)
Supplement: Supplementary file 4 — Description of Additional Supplementary Files [file 42003_2022_3469_MOESM4_ESM.pdf]

Description of Additional Supplementary Files

File name: Supplementary Data 1
Description: The source data for Table S1, Figure 2a-e, 3b, 3g, 3h, S3, S4 and S5 in the paper.
